# A novel small molecule RK-019 inhibits *FGFR2*-amplification gastric cancer cell proliferation and induces apoptosis *in vitro* and *in vivo*

**DOI:** 10.3389/fphar.2022.998199

**Published:** 2022-09-21

**Authors:** Jun Zeng, Kai Ran, Xinyue Li, Longyue Tao, Qiwei Wang, Jiangtao Ren, Rong Hu, Yongxia Zhu, Zhihao Liu, Luoting Yu

**Affiliations:** ^1^ State Key Laboratory of Biotherapy and Cancer Center, Sichuan University and Collaborative Innovation Center for Biotherapy, West China Hospital, Sichuan University, Chengdu, China; ^2^ College of Pharmacy, National and Local Joint Engineering Research Center of Targeted and Innovative Therapeutics, Chongqing Key Laboratory of Kinase Modulators as Innovative Medicine, Chongqing University of Arts and Sciences, Chongqing, China; ^3^ Department of Clinical Pharmacy, Sichuan Cancer Hospital and Institute, Sichuan Cancer Center, School of Medicine, University of Electronic Science and Technology of China, Chengdu, China; ^4^ Research Laboratory of Emergency Medicine, Department of Emergency Medicine, West China Hospital, Sichuan University, Chengdu, China

**Keywords:** gastric cancer, receptor tyrosine kinase, antineoplastic agents, FGFRs, cell apoptosis

## Abstract

Gastric cancer (GC) is one of the most malignant cancers and is estimated to be fifth in incidence ratio and the third leading cause of cancer death worldwide. Despite advances in GC treatment, poor prognosis and low survival rate necessitate the development of novel treatment options. Fibroblast growth factor receptors (FGFRs) have been suggested to be potential targets for GC treatment. In this study, we report a novel selective FGFR inhibitor, RK-019, with a pyrido [1, 2-a] pyrimidinone skeleton. *In vitro*, RK-019 showed excellent FGFR1-4 inhibitory activities and strong anti-proliferative effects against *FGFR2*-amplification (*FGFR2*-amp) GC cells, including SNU-16 and KATO III cells. Treatment with RK-019 suppressed phosphorylation of FGFR and its downstream pathway proteins, such as FRS2, PLCγ, AKT, and Erk, resulting in cell cycle arrest and induction of apoptosis. Furthermore, daily oral administration of RK-019 could attenuate tumor xenograft growth with no adverse effects. Here, we reported a novel specific FGFR inhibitor, RK-019, with potent anti-FGFR2-amp GC activity both *in vitro* and *in vivo*.

## Introduction

Gastric cancer (GC) is one of the most malignant cancers and is estimated to be fifth in incidence ratio and the third leading cause of cancer death worldwide (Sung et al., 2021). Patients with early-stage GC are mostly asymptomatic, and most diagnoses are made at the medium or advanced stage (Takahashi et al., 2013). However, at these stages, chemotherapy, radiotherapy, and surgical treatment efficacies are very low (Van Cutsem et al., 2016). Hence, developing new therapeutic strategies with high efficacy and low toxicity for the treatment of advanced GC is crucial.

Fibroblast growth factor receptors (FGFR1-4) belong to the receptor tyrosine kinase family that bind to numerous fibroblast growth factor (FGF) members (Ornitz and Itoh, 2001; Beenken and Mohammadi, 2009; Belov and Mohammadi, 2013). Extracellular ligand FGFs bind to FGFRs and induce FGFR dimerization and then initiate downstream intracellular signaling cascade pathways, including those involving Ras-Raf-Erk, PI3K-AKT-mTOR, and PLCγ/Ca^2+^ (Sarabipour and Hristova, 2016). Moreover, the JAK2-STAT pathway can be activated by FGFRs in certain cellular contexts (Deo et al., 2002; Cerliani et al., 2011; Li et al., 2019). Furthermore, by regulating signaling cascade pathways, FGFRs mediate physiological processes, such as development progress, cell proliferation, differentiation, and angiogenesis (Eswarakumar et al., 2005; Fukumoto, 2008; Brooks et al., 2012). Anomalously activated FGFR signaling due to FGFR mutations, amplifications, and translocations is involved in tumorigenesis and progression of cancers, including breast cancer, prostate cancer, GC, urothelial cancer, and cholangiocarcinoma (Grose and Dickson, 2005; Katoh, 2010; Lamont et al., 2011; Jain et al., 2018).

Studies indicated that FGFR1 overexpression facilitates peritoneal diffusion *via* epithelial-to-mesenchymal transition (EMT) in GC (Shimizu et al., 2018). Overexpression of FGFR1 in GC tissue samples was correlated with EphA4 protein expression whose synergy promoted GC development (Oki et al., 2008). In addition, FGFR2 is considered to play an important role in GC, especially in GC with chromosomal instability (Network, 2014). FGFR2 overexpression was identified in 60% of patients with GC, and *FGFR2*-amplification (*FGFR2*-amp) was found to occur in approximately 2–15% of these patients (Matsumoto et al., 2012; Su et al., 2014; Han et al., 2015; Ahn et al., 2016; Jia et al., 2016; Kim et al., 2019b). *FGFR2* expression level, amplification, and mutations are associated with drug resistance and prognosis (Matsumoto et al., 2012; Tokunaga et al., 2016; Hosoda et al., 2017). Furthermore, *FGFR2*-amp is an adverse prognostic factor in GC patients. Compared to *FGFR2*-unamplified GC, *FGFR2*-amp is significantly associated with lymph node metastasis and worse survival rate (Kim et al., 2019a). Besides, FGFR3 and FGFR4 expression levels and mutations are associated with poor prognosis and drug resistance, inhibition of cell proliferation signals, and induction of apoptosis (Jang et al., 2001; Ye et al., 2012; Ye et al., 2013; Piro et al., 2016). Evidence suggests that *FGFR* knockdown or inhibition selectively inhibits GC cell line growth (Katoh, 2010; Jang et al., 2017; Zhang et al., 2019). Hence, pharmacological targeting of FGF/FGFRs signaling pathways may be effective for the treatment of FGFRs-altered GC.

Based on biochemistry and cell-based screening from our laboratory’s bioactive compound library, we found a novel FGFR inhibitor RK-019. In this study, we evaluated the anti-neoplastic activity of RK-019 both *in vitro* and *in vivo*. RK-019 showed excellent inhibition and great selectivity against FGFR family kinases, strong anti-proliferative effects, and anti-metastasis on *FGFR2*-amp GC cells. Therefore, RK-019 might be an effective treatment agent against *FGFR2*-amp GC, making it a candidate drug for GC treatment.

## Materials and methods

### Materials

RK-019 was synthesized in our laboratory (State Key Laboratory of Biotherapy, Sichuan University, Chengdu, China). RK-019 was dissolved in dimethyl sulfoxide (DMSO) from Sigma (D8418, St Louis, Mo, United States) at a stock concentration of 40 mM and stored in a -80°C refrigerator. The working solution was diluted to 100 nM, 10 nM, 5 nM, 1 nM, and 0.1 nM by DMSO and stored in a -20°C refrigerator. 3-(4,5-Dimethyl-2-thiazolyl)-2,5-diphenyl-2-H-tetra-zolium bromide (MTT) was purchased from CSNpharm (CSN12440, Shanghai, China). A PE Annexin V Apoptosis Detection Kit was purchased from BD Biosciences (559763, Franklin, NJ, United States). Antibodies: FGFR2 (23328), p-FRS2 (3864), PLCγ (5690), p-PLCγ (14008), AKT (4691), p-AKT (4060), Erk (4695), p-Erk (4370), CDK 2 (18048), CDK 4 (12790), CDK 6 (13331), Cyclin D1 (55506), Cyclin E (4136), p27 (3686), Caspase 3 (9662), and Cleaved-caspase 3 (9661) were purchased from Cell Signaling Technology (Beverly, MA, United States). p-FGFR2^T653/T654^ (AF8210), JAK2 (AF6022), p-JAK2^T1007^ (AF3022), STAT3 (AF6294), and p-STAT3^T705^ (AF3293) were purchased from Affinity Biosciences (Changzhou, China). MMP-2 (CY7164) and MMP-9 (CY5205) were purchased from Abways (Shanghai, China). FRS2 (R26776) and β-actin (200068-8F10) are from Zen-bio (Chengdu, China).

### Synthesis of RK-019

#### Synthesis route diagrammed in Figure 1C

Step i: (*E*)-5-(((5-bromopyridin-2-yl)imino)methyl)-2,2-dimethyl-1,3-dioxane-4,6-dione (2). A mixture of triethyl orthoformate (8.5 g, 80 mmol) and 2,2-dimethyl-1,3-dioxane-4,6-dione (11.5 g, 80 mmol) was heated at 60°C for 2 h. Then, a solution of 5-bromopyridin-2-amine (1, 13.8 g, 80 mmol) in EtOH (80 ml) was added slowly and the resulting reaction mixture was stirred at 60°C for additional 2 h. Upon completion of the reaction, the mixture was cooled to room temperature. The precipitate was filtered and the filter cake was washed with a small amount of EtOH and dried in a vacuum oven to afford 2 as white solid (16.6g, 64%). ^1^H NMR (400 MHz, CDCl_3_): δ 11.39–11.24 (m, 1 H), 9.38–9.28 (m, 1 H), 8.47 (d, *J* = 2.4 Hz, 1H), 7.86 (dd, *J* = 8.5, 2.4 Hz, 1H), 6.95 (d, *J* = 8.5 Hz, 1H), and 1.76 (s, 6H). ESI-MS: C_12_H_11_BrN_2_O_4_, MS (ESI) *m/z* 327.1 [M + H]^+^.

**FIGURE 1:**
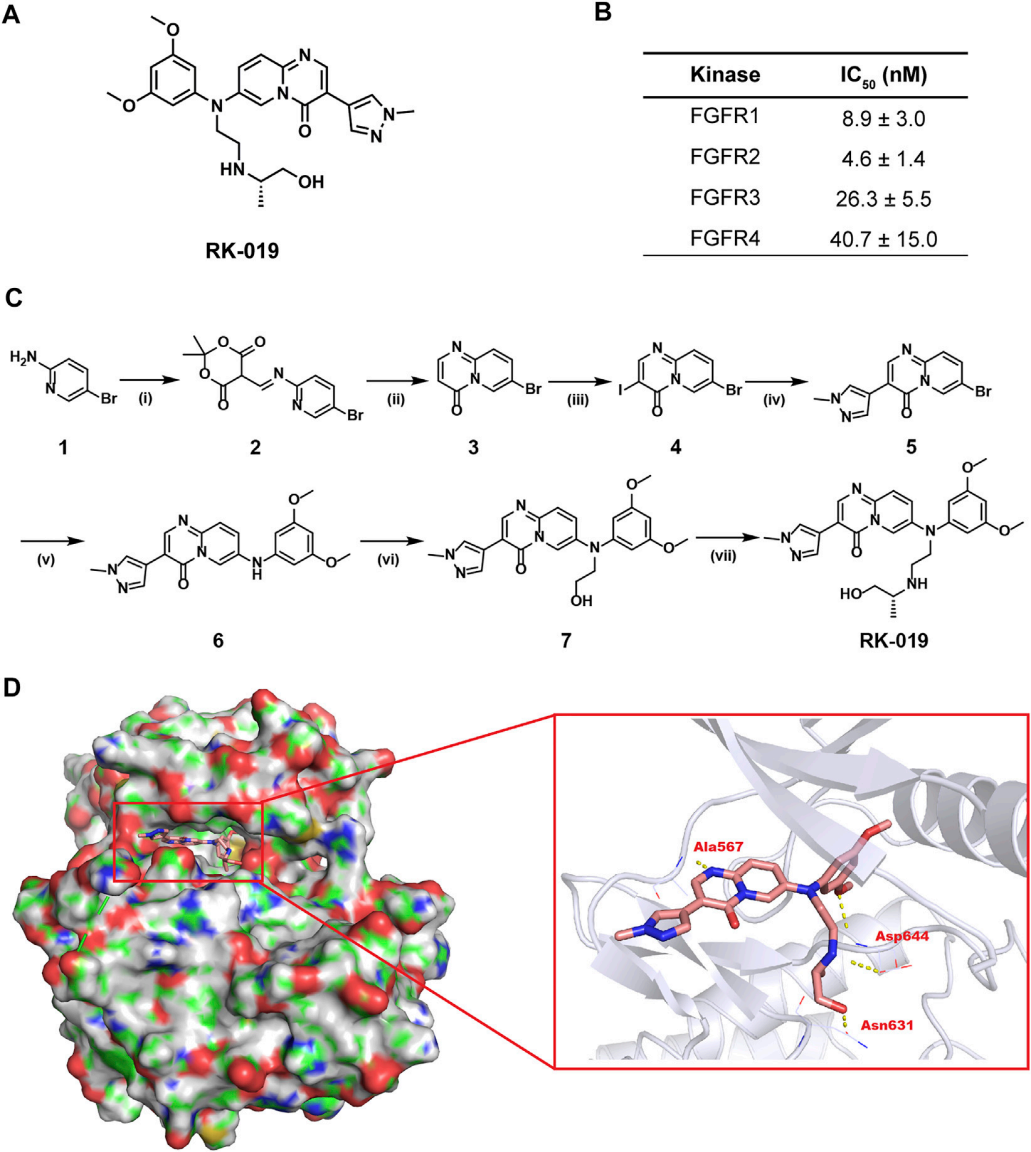
Development of RK-019. **(A)** Chemical structure of RK-019. **(B)** IC_50_ values of RK-019 against kinases FGFR 1–4. **(C)** Synthesis route of RK-019. Reagents and conditions: (i) Triethyl orthoformate, 2,2-dimethyl-1,3-dioxane-4,6-dione, EtOH, reflux; (ii) Diphenyl oxide, 220°C; (iii) NIS, DMF, 80°C; (iv) Boronic acids or boronate esters, Pd (dppf)_2_Cl_2_, K_2_CO_3_, dioxane, H_2_O, 100°C; (v) 3,5-Dimethoxyaniline, Pd_2_ (dba)_3_, 2,2′-bis(diphenylphosphino)-1,1′-dinaphthalene, Cs_2_CO_3_, toluene, 100°C; (vi) (a) 2-(tert-Butyldimethylsilyloxy)bromoethane, NaH, DMF, 5°C–r.t.; **(b)** TBAF, THF, r. t.; and (vii) (a) MsCl, Et_3_N, CH_2_Cl_2_, 0°C; (b) (R)-1-(aminomethyl)ethanol, CH_3_CN, 100°C. **(D)** Docking results of RK-019 on FGFR2 protein (PDB ID: 6AGX).

Step ii: 7-bromo-4*H*-pyrido [1,2-a]pyrimidin-4-one (3). Ph_2_O (200 ml) was heated to 220°C, then 2 (16.6 g, 51 mmol) was slowly added into the solution. The mixture was stirred at 220°C for 30 min. TLC detected that the reaction was completed. The mixture was cooled and purified by column chromatography with petroleum ether/ethyl acetate (4:1) to afford 3 (10.7g, 93%). ^1^H NMR (400 MHz, DMSO-*d*_*6*_): δ 9.03 (d, *J* = 2.2 Hz, 1H), 8.32 (d, *J* = 6.4 Hz, 1H), 8.08 (dd, *J* = 9.4, 2.2 Hz, 1H), 7.65 (d, *J* = 9.4 Hz, 1H), and 6.46 (d, *J* = 6.4 Hz, 1H). ESI-MS: C_8_H_5_BrN_2_O, MS (ESI) *m/z* 225.2 [M + H]^+^.

Step iii: 7-bromo-3-iodo-4*H*-pyrido [1,2-a]pyrimidin-4-one (4). To a stirred solution of 3 (10.2 g, 45 mmol) in DMF (50 ml) was added NIS (13.6g, 60 mmol), the mixture was stirred at 80°C for 5 h. Upon completion of the reaction, the reaction mixture was cooled to room temperature, and then added with H_2_O (50 ml) under stirring. The precipitate was collected by filtration, washed with water dried to a constant weight to afford 4 (15.0 g, 95%).^1^H NMR (400 MHz, DMSO-*d*_*6*_): δ 9.02 (d, *J* = 2.0 Hz, 1H), 8.75 (s, 1H), 8.13 (dd, *J* = 9.4, 2.0 Hz, 1H), 7.67 (d, *J* = 9.4 Hz, 1H). MS (ESI) *m/z* 351.0 [M + H]^+^.

Step iv: 7-bromo-3-(1-methyl-1H-pyrazol-4-yl)-4H-pyrido [1,2-a]pyrimidin-4-one (5). 4 (14.1 g, 40 mmol) and 1-methyl-4-(4,4,5,5-tetramethyl-1,3,2-dioxaborolan-2-yl)-1H-pyrazole (8.3 g, 40 mmol), Na_2_CO_3_ (8.5 g, 80 mmol) were dissolved in dioxane (100 ml) and H_2_O (25 ml). The suspension was degassed under nitrogen bubbling for 10 min before Pd (dppf)_2_Cl_2_ (2.9 g, 4 mmol) was added. The reaction mixture was heated to 100°C for 5 h, and then diluted with ethyl acetate. The solution was washed with water and brine successively, dried over anhydrous sodium sulfate, and concentrated in vacuum. The residue was purified *via* silica gel chromatography with petroleum ether/ethyl acetate (2:1) to afford 5 (8.1 g, 66%). ^1^H NMR (400 MHz, DMSO-*d*_*6*_): δ 8.79–8.68 (m, 2H), 8.32 (s, 1H), 8.10 (s, 1H), 7.78 (dd, *J* = 9.6, 2.6 Hz, 1H), 7.69 (d, *J* = 9.6 Hz, 1H).

Step v: 7-((3,5-dimethoxyphenyl)amino)-3-(1-methyl-1*H*-pyrazol-4-yl)-4*H*-pyrido [1,2-a]pyrimidin-4-one (6). A mixture of 5 (8.1 g, 26.3 mmol), 3,5-dimethoxyaniline (4.8 g, 31.6 mmol), Pd_2_ (dba)_3_ (2.4 g, 2.63 mmol), (±)-BINAP (2.5 g, 3.95 mmol), and cesium carbonate (12.9 g, 39.5 mmol) in anhydrous toluene (120 ml) was degassed with N_2_ for 10 min. The reaction was heated to 100°C overnight under N_2_, and then cooled to room temperature. The mixture was filtrated through a Celite and the filter cake was washed with DCM/MeOH (10/1). After concentration of the filtrate, the residue was purified by column chromatography with methyl allylchloride/methanol (20:1) to give 6 (7.1 g, 56%).^1^H NMR (400 MHz, DMSO-*d*_*6*_): δ 10.70 (s, 1H), 8.67 (d, *J* = 2.6 Hz, 1H), 8.48 (s, 1H), 8.23 (s, 1H), 7.89 (s, 1H), 7.36 (d, *J* = 9.6 Hz, 1H), 7.25 (dd, *J* = 9.6, 2.6 Hz, 1H), 6.28–6.16 (m, 3H), 3.86 (s, 3H), and 3.74 (s, 6H).

Step vi: 7-((3,5-dimethoxyphenyl) (2-hydroxyethyl)amino)-3-(1-methyl-1*H*-pyrazol-4-yl)-4*H*-pyrido [1,2-a]pyrimidin-4-one (7). To a solution of 6 (4.0 g, 10.6 mmol) in DMF (60 ml) was added sodium hydride (60% dispersion in mineral oil, 933 mg, 23.32 mmol) slowly at 0 °C under argon. After being stirred at this temperature for 30 min, (2-bromoethoxy) (tert-butyl)dimethylsilane (4.6 ml, 21.2 mmol) was added and then the reaction mixture was stirred at room temperature overnight. Following this time, the reaction mixture was diluted with water and then extracted with ethyl acetate twice. The combined extracts were washed with water and brine, dried over anhydrous Na_2_SO_4_, and concentrated under reduced pressure. The residue was dissolved in tetrahydrofuran (20 ml) and tetrabutylammonium fluoride (1 M in tetrahydrofuran, 21.2 ml, 21.2 mmol) was added. The reaction mixture was stirred at room temperature overnight. Following this time, the reaction mixture was concentrated under vacuum and the resulting residue was purified by column chromatography with methyl allylchloride/methanol (10:1) to give the title compound 7 as yellow solid (2.5g, 56%). ^1^H NMR (400 MHz, DMSO-*d*_*6*_): δ 8.77–8.71 (m, 2H), 8.38 (s, 1H), 8.12 (s, 1H), 7.68 (dd, *J* = 9.6, 2.6 Hz, 1H), 7.61 (d, *J* = 9.6 Hz, 1H), 6.43–6.32 (m, 3H), 4.96 (t, *J* = 5.0 Hz, 1H), 3.93–3.83 (m, 5H), 3.71 (s, 6H), and 3.68–3.61 (m, 2H). HRMS: calculated for C_22_H_23_N_5_O_4_ [(M + H)^+^], 422.1824; found 422.1821.

Step vii: (*R*)-7-((3,5-dimethoxyphenyl) (2-((1-hydroxypropan-2-yl)amino)ethyl)amino)-3-(1-methyl-1*H*-pyrazol-4-yl)-4*H*-pyrido [1,2-a]pyrimidin-4-one (RK-019). To a mixture of 7 (2.5 g, 5.9 mmol) and Et_3_N (2.1 ml, 14.75 mmol) in DCM (30 ml) was added methanesulfonyl chloride (0.917 ml, 11.8 mmol) dropwise at 0°C under argon. The reaction mixture was stirred at room temperature for 2 h. After completion (monitored by TLC), the reaction mixture was quenched with water, then extracted with DCM twice. The combined extracts were washed with water and brine successively, dried over anhydrous Na_2_SO_4_, and concentrated under vacuum. The residue was purified by column chromatography to give the intermediate (2.2 g). A mixture of the intermediate (2.2 g, 4.4 mmol) and (*R*)-1-(aminomethyl)ethanol (3.3 g, 44 mmol) in acetonitrile (44 ml) was heated at 100°C overnight. After completion (monitored by TLC), the reaction mixture was concentrated under vacuum and the residue was purified on TLC-preparative plates with methyl allylchloride/methanol (10:1) to afford the desired product RK-019 as light-yellow solid (366 mg, 18%), mp.144–150°C. ^1^H NMR (400 MHz, DMSO-d6): δ 8.73 (s, 1H), 8.66 (d, J = 2.5 Hz, 1H), 8.38 (s, 1H), 8.12 (s, 1H), 7.66 (dd, J = 9.6, 2.5 Hz, 1H), 7.61 (d, J = 9.6 Hz, 1H), 6.38 (d, J = 2.1 Hz, 2H), 6.31 (d, J = 2.1 Hz, 1H), 4.51–4.43 (m, 1H), 3.89 (s, 3H), 3.84 (t, J = 6.5 Hz, 2H), 3.72 (s, 6H), 3.29–3.20 (m, 3H), 2.86–2.75 (m, 2H), 2.63–2.57 (m, 1H), and 0.90 (d, J = 6.2 Hz, 3H). ^13^C NMR (101 MHz, DMSO-*d*_6_) δ 161.88, 149.53, 148.44, 147.50, 143.96, 140.08, 138.25, 135.93, 131.16, 129.47, 121.78, 120.85, 109.54, 104.32, 97.49, 55.76, 52.78, 48.55, 44.28, and 23.31. HRMS: calculated for C_25_H_30_N_6_O_4_ [(M + H) ^+^], 479.2403; found 479.2405.

### Molecular docking

The 3D structure of FGFR2 was downloaded from the PDB (http://www.rcsb.org/, PDB ID: FGFR1: 5EW8, FGFR2: 6AGX, FGFR3: 6LVM, and FGFR4: 6NVK). The protein was prepared with discovery studio 3.1. Molecule RK-019 was built with ChemBio3D and optimized at the molecular mechanical level. Then, RK-019 was docked to the binding site of JNJ42756493 by employing a protein-ligand docking program GOLD 2.5, respectively. Scoring function GOLDSCORE was used for exhaustive searching, solid body optimizing, and interaction scoring. The final results for molecular docking were visualized by using the PyMol program.

### Kinase inhibition and selectivity assay

Median-inhibitory concentration (IC_50_) and inhibition ratio of kinases by RK-019 was assessed by Eurofins Discovery Services (Dundee, United Kingdom) using the ATP-site competition binding assay *in vitro*. Firstly, gradient concentration of RK-019 against FGFRs, including FGFR1, FGFR2, FGFR3, and FGFR4, were tested. IC_50_ value was calculate by the GraphPad Prism software (v8.4.3, GraphPad Software, California, United States). Then, to measure the selectivity prolife of RK-019, a panel of 422 recombinant human kinases was screened at a concentration of 1 µM RK-019. Kinase MAP was drawn by webtools from Cell Signaling Technology, Inc. (www.cellsignal.com).

### Cell lines

SNU-16, KATO III, AZ 521, MGC 80–3, HGC 27, N87, AGS, NUGC-4, GT 39, MKN 45, BGC823, and GES-1 were purchased from BeNa Culture Collection (Beijing, China). HGC27, AGS, NUGC-4, and GT39 cell lines were cultured in DMEM (L110KJ, BasalMedia, Shanghai, China) with 10% of fetal bovine serum (900–108, Gemini, California, USA). SNU-16, AZ521, MGC 80–3, N87, MKN45, BGC823, and GES-1cell lines were cultured in RPMI-1640 medium (L210KJ, BasalMedia) with 10% of fetal bovine serum. KATO III cell was cultured in IMDM medium (L610KJ, BasalMedia) with 20% of fetal bovine serum. All the culture medium was contained with 1% antibiotic (30–002-CI, Corning, Corning, NY, United States). All the cell lines were maintained in 5% CO_2_ condition at 37 °C.

### Cell viability assay

For suspension and mixed suspension adherent cells, the cells were seeded in 96-well plate with different cell density, 0.5×10^4^ per well for 96 h treatment, 1×10^4^ per well for 72 h treatment, 2×10^4^ per well for 48 h treatment, and 5×10^4^ per well for 24 h treatment. Different doses of RK-019 were treated as soon as the cell seeded in 96-well plates. For adherent cells, the cells were seeded in a 96-well plate with 3×10^3^ per well and cultured overnight followed by administrated different doses of RK-019. After indicated time treatment, MTT solution at a final concentration of 0.5 mg/ml was added and incubated 2–4 h at 37°C. Formazan formed by the living cells was dissolved with DMSO and the absorbance was measured using a microplate spectrophotometer (MultiskanFC, ThermoFisher, Waltham, MA, United States) at 570 nm. IC_50_ value of RK-019 was calculated by the Graphpad Prism software.

### Quantitative reverse transcription PCR (qRT-PCR)

The GC cell total RNAs were extracted by TRIzol (15596026, ThermoFisher). RNA was reversed transcribed into cDNA by using HiScript III RT SuperMix for qPCR (R323-01, Vazyme, Nanjing, China) according to the manufacturer’s protocol. qRT-PCR for *FGFR2* gene expression was carried out by ChamQ universal SYBR qPCR Master Mix (Q711-02, Vazyme) on Bio-Rad CFX96 Realtime PCR system. The PCR primer for *FGFR1*: forward 5‘- GCT​ACA​AGG​TCC​GTT​ATG​C -3’ and reverse 5′- CAA​TGC​AGG​TGT​AGT​TGC​C -3’, *FGFR2*: forward 5‘- GGT​GGC​TGA​AAA​ACG​GGA​AG -3’ and reverse 5′- AGA​TGG​GAC​CAC​ACT​TTC​CAT​A -3’, *FGFR3*: forward 5‘- TGC​GTC​GTG​GAG​AAC​AAG​TTT -3’ and reverse 5′- GCA​CGG​TAA​CGT​AGG​GTG​TG -3’, *FGFR4*: forward 5‘- CCA​TAG​GGA​CCC​CTC​GAA​TAG -3’ and reverse 5′- CAG​CGG​AAC​TTG​ACG​GTG​T -3’, and *ACTB*: forward 5‘- CAC​CAT​TGG​CAA​TGA​GCG​GTT​C -3’ and reverse 5′- AGG​TCT​TTG​CGG​ATG​TCC​ACG​T -3’. The gene expression analysis was conducted by the ΔΔCq method.

### Cell counting assay

SNU-16 and KATO III were seeded in 6-well plated with 1×10^5^ per well. RK-019 was treated as soon as the cells seeded in the 6-well plates. After indicated time, the images were captured using an inverted microscope. Then, the cells in the captured well were resuspended and live cell numbers were determined by the trypan blue (BL627A, Biosharp, Hefei, China) method.

### Edu staining assay

For Edu staining assay, 1×10^5^ cells were seeded in 24-well plates. RK-019 and DMSO were treated as soon as the cells seeded into the plates. After 24 h, the cells were incubated with the medium contained 10 µM Edu for 2 h. After that, the cells were collected and fixated to slide, and then stained with the Cell-Light EdU Apollo488 *In Vitro* Kit (C10310, Ribobio, Guangzhou, China). After staining, the samples were photographed and analyzed by the ImageJ software (v1.53a, Wayne Rasband, United States).

### Cell cycle and apoptosis analysis

Cell cycle and apoptosis assay were both measured by flow cytometry (FCM). The data were analyzed by the NovoExpress software (v1.4.0, Agilent, Santa Clara, CA, United States). For cell cycle assay, the cells were treated by different concentrations of RK-019 for 24 h, then were harvested and washed twice with cold phosphate buffered solution (PBS), followed by fixed with 75% ethanol overnight. Before test, the cells were washed twice with cold PBS and stained with PI stanning solution using cell cycle detecting kit (KGA512, KeyGEN, Nanjing, China), and then measured by FCM. For apoptosis assay, the cells were harvested after treated with different concentrations of RK-019 for 24 h, and stained with a PE Annexin V Apoptosis Detection Kit according to the manufacturer’s protocol followed by detected using FCM.

### Transwell migration and invasion assay

In the migration assay, 1×10^5^ cells were resuspended in a serum-free medium, then added into the upper chamber, meanwhile the medium containing 10% FBS was added at the bottom with 100 nM RK-019. After 24 h incubation, the migration cells on the filters were fixed with 4% paraformldehyde and washed with PBS, then stained with crystal violet solution for 20 min. In the invasion assay, the upper surface of the transwell was coated with Matrigel (356234, Corning) for 30 min at 37°C until Matrigel was solidified. 1×10^5^ cells were resuspended in a serum-free medium, then added into the upper chamber, meanwhile the medium containing 10% FBS was added at the bottom with 100 nM RK-019.After 24 h , the invasion cells on the filters were hatched and fixed, then stained with crystal violet.

### Western blot analysis

The cells treated with RK-019 at indicated concentration for 24 h were harvested and lysed in 1× RIPA buffer (20e188, MilliporeSigma, Burlington, MA, United States), which contained protease inhibitor cocktail (B14001, Bimake, Houston, TX, United States) and phosphatase inhibitor cocktail (B15001, Bimake), for 30 min and equalized by concentration of the total protein before loading. Using sodium dodecyl sulphate - polyacrylamide gel electrophoresis (SDS-PAGE) to separate the protein sample and transfer to PVDF membranes (ISEQ00010, MilliporeSigma). The membranes were incubated with relevant primary antibody and corresponding HRP-labeled secondary antibody. Then, chemiluminescence was used to detect target bands. ImageJ software were used to make the gradation analysis.

In western blot, results analysis of relative expression quantification level, data were firstly calculated according to the following formular: Protein Relative expression data = G_t_/G_i_. G_t_ means the gradation level of target protein band and G_i_ means the gradation level of internal reference protein band. Then, the relative expression level can be calculated by the following formular: Protein Relative expression level = D_t_/D_c_ ×100%. D_t_ means the protein relative expression data of target group and D_c_ means the protein relative expression data of control group.

In western blot, results analysis of relative phosphorylation quantification level, data were firstly calculated according to the following formular: Protein Relative phosphorylation data = G_p_/G_up_. G_p_ means the gradation level of target phosphorylation protein band and G_up_ means the gradation level of target total protein band. Then, the relative phosphorylation level can be calculated by the following formular: Protein Relative expression level = P_t_/P_c_ ×100%. P_t_ means the relative phosphorylation data or target group and P_c_ means the relative phosphorylation data of control group.

### *In vivo* PK assay

All animal experiments in this study have been approved by the Institutional Animal Care and Treatment Committee of Sichuan University in China and were carried out in accordance with the approved guidelines. Sprague-Dawley rats (weight 180–200 g, Beijing HJF bioscience, Beijing, China) were used in this experiment and maintained in a specific-pathogen-free (SPF) condition facility. RK-019 was dissolve in ethanol first, followed by mixed with Kolliphor EL, then filled with saline to the calculated volume. The formula was 12.5% ethanol, 12.5% Kolliphor EL, and 75% saline. RK-019 was administrated *via* i. v. 3 mg/kg or p. o. 30 mg/kg. After 5, 10, 15, 30, 60, 120, 240, 360, 600, and 1,440 min of administration, the blood samples were collected. Plasma fraction was obtained by centrifuged blood, then deproteinized with methanol containing an internal standard. The compound concentrations of the target compound in the supernatant were measured by liquid chromatography–mass spectrometry/mass spectrometry (LC-MS/MS).

### Subcutaneous xenograft model

NOD/SCID mice (6-week-old, weight 18–20 g, Beijing HJF bioscience) were used in this experiment and maintained in a specific-pathogen-free (SPF) condition facility. SNU-16 Cells were resuspended in PBS mixed with Matrigel (1:1) and adjust the density to 1×10^7^ per mice (100 μL). The cell suspensions were inoculated subcutaneously on the right flank of the mice. When the mean tumor volume reached approximately 200 mm^3^, the mice were divided into four groups (five mice each group): i. Vehicle group, ii. RK-019 15 mg/kg group, iii. RK-019 30 mg/kg group, and iv. RK-019 45 mg/kg group. RK-019 was dissolved in same formula as the *in vivo* PK study. RK-019 was administrated once *per* day by oral gavage for 21 days. Tumor volumes were measured three times *per* week. The tumor volume was measured by a caliper and calculated according to the following formula:

$$Tumor\;volume=\frac{Major\;axis\times {Miner\;axis}^{2}}{2}.$$

At the end of administration, the mice were executed euthanasia, then tumors, blood, and organs were extracted. The complete blood count (CBC), serum phosphorus analysis, and blood biochemical analysis were completed by West China Frontier Pharma Tech (Chengdu, China). The pathological section and IHC stanning was completed by Servicebio (Wuhan, China). The tumor growth inhibition (TGI) values were calculated with the following formula:

$$TGI=\left(1-\frac{T_{n}-T_{0}}{C_{n}-C_{0}}\right)\times 100\%.$$

T_n_ and T_0_ represent average tumor volume before treatment and that of day n after treatment in the treatment group. C_n_ and C_0_ represent average tumor volume before treatment and that of day n after treatment in the vehicle group. In this study, n is 21st day.

### Statistical analyses

All data were analyzed by the GraphPad Prism 8.4.3 (GraphPad Software, California, United States), and was shown as mean value ±SD or SEM, details will be illustrated in the figure legends. Dose-effect curve analysis (IC_50_ calculation) was performed by the Graphpad Prism software. First, concentration value was converted to log value, followed by non-linear regression analyze which is l*og(inhibitor)* vs. *normalized response—Variable slope*, was performed to calculate the IC_50_ values. The statistically significant *p* values were calculated by student’s t-test or two-way ANOVA and were shown in graphics which were labeled as follows: *p < 0.05; **p < 0.01; ***p < 0.001.

## Result

### Development of RK-019

In this study, we identified a novel small molecule pan-FGFR inhibitor, RK-019, whose chemical structure was named as (*R*)-7-((3,5-dimethoxyphenyl) (2-((1-hydroxypropan-2-yl)amino)ethyl)amino)-3-(1-methyl-1*H*-pyrazol-4-yl)-4*H*-pyrido [1,2-a]pyrimidin-4-one (Figure 1A). The method of synthesis of RK-019 is described in detail in the materials and methods section (Figure 1C). Briefly, RK-019 was synthesized from 5-bromopyridin-2-amine 1) with imidization, cyclization, iodination, and Suzuki coupling of 4 with 1-methyl-4-pyrazole boronic acid pinacol ester, followed by Buchwald coupling to get 6. Finally, nucleophilic substitution with 6 and 2-isopropylaminoethylchloride and deprotection by hydrochloric acid produced RK-019. ^1^H-NMR, ^13^C-NMR, and HRMS spectrometry results were shown in Supplementary Figure S3.

Molecular docking was conducted to model the binding of RK-019 in the ATP pocket using a reported crystal structure of FGFR kinase domain (PDB ID: 6AGX). As shown in Figure 1D, RK-019 could fit well in the ATP pocket of FGFR2 and the *N*-methyl pyrazole group could extend into the solvent region. RK-019 maintained H-bond interactions with FGFR2 at the backbone NH of Ala567 in the hinge region. The methoxyphenyl motif occupied the hydrophobic region in the ATP pocket and its methoxy group formed a H-bond with the NH of Asp644. The 2-amino-propanol group can interact with the main carbonyl group of Asp644 and Asn631 to form two hydrogen bonds. Overall, RK-019 could fit well into the ATP pocket of FGFR2. Similarity, RK-019 could also fit well in FGFR1 (PDB ID: 5EW8), FGFR3 (PDB ID: 6LVM), and FGFR4 (PDB ID: 6NVK) ATP binding pocket (Supplementary Figure S1). Then, the ATP-based kinase activity assay provided by kinase profile service (Eurofins Discovery) revealed that RK-019 was sufficient to inhibit the FGFR family kinase activity, with the IC_50_ of 9.1 nM (FGFR1), 4.6 nM (FGFR2), 26.3 nM (FGFR3), and 40.7 nM (FGFR4) (Figure 1B).

### Kinase selectivity profile of RK-019

**FIGURE 2:**
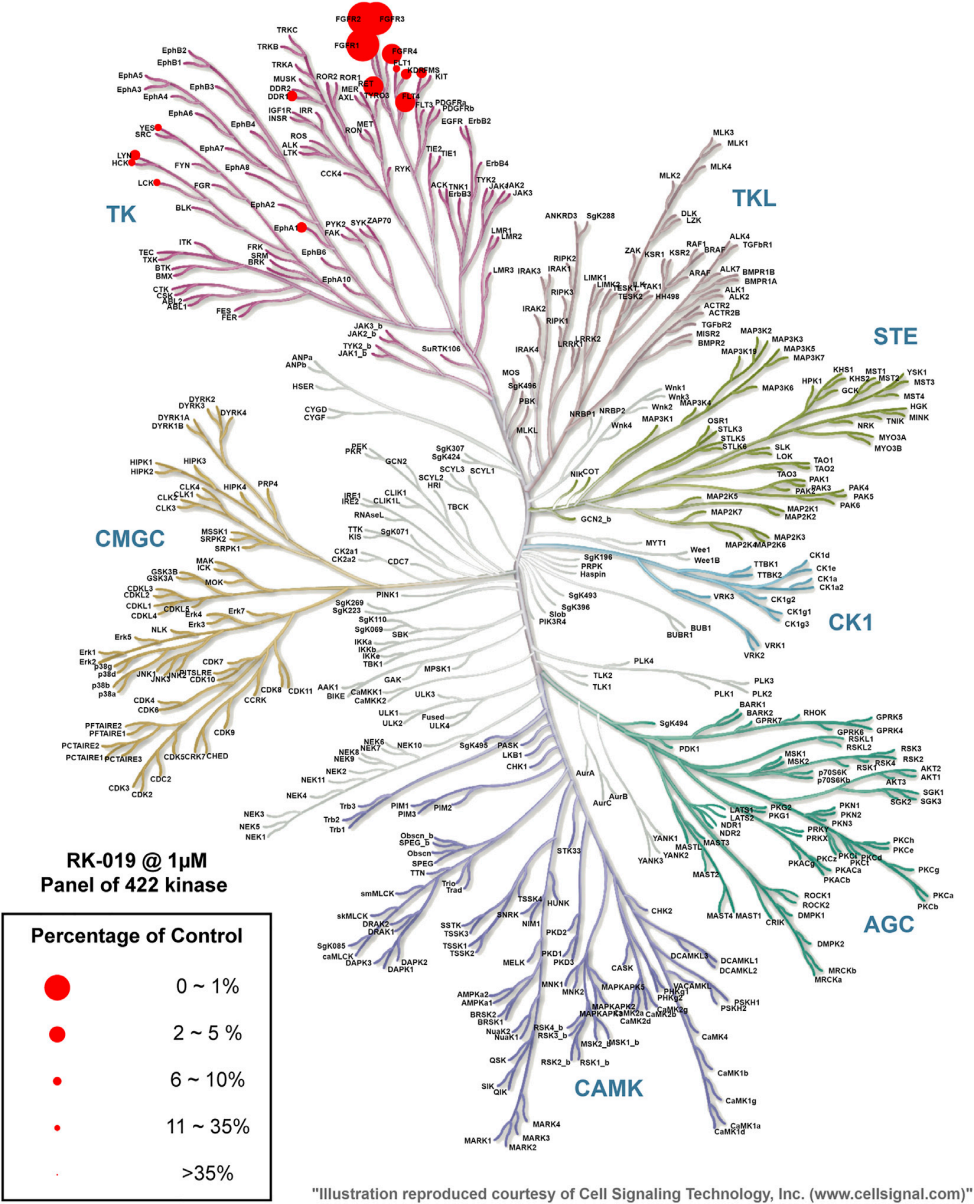
Kinase selectivity profile of RK-019. Measurement of 422 kinases was performed at 1 μM of RK-019. Each kinase was measured once. Data were cutoff on 35% kinase activity compared to the DMSO group. The TREE spot image was mapped with the KinMap software tool provided by Cell Signaling Technology, Inc. (www.cellsignal.com). The percentage of control means remaining active kinase percentage.

To further investigate the kinase selectivity, the kinase inhibitory profile of RK-019 was determined against a diverse panel of 422 recombinant human kinases from Eurofins Discovery by the ATP-site competition binding assay at a concentration of 1 µM (Supplementary Table S1). The kinase inhibition results were depicted in a kinase MAP at a 35% activity cutoff of the DMSO control (Figure 2). Of these, only seven hits (FGFR1, FGFR2, FGFR3, FGFR4, FLT4, RET, and LYN) showed over 90% of inhibition. A further enzyme activity assay was performed to determine the IC_50_ values of these seven hits. As results shown, RK-019 showed a great selectivity to the FGFRs family kinase, which indicated that the molecule was a potent and selective pan-FGFR inhibitor.

### Anti-proliferation effects of RK-019 against *FGFR2*-amp GC cell lines

**FIGURE 3:**
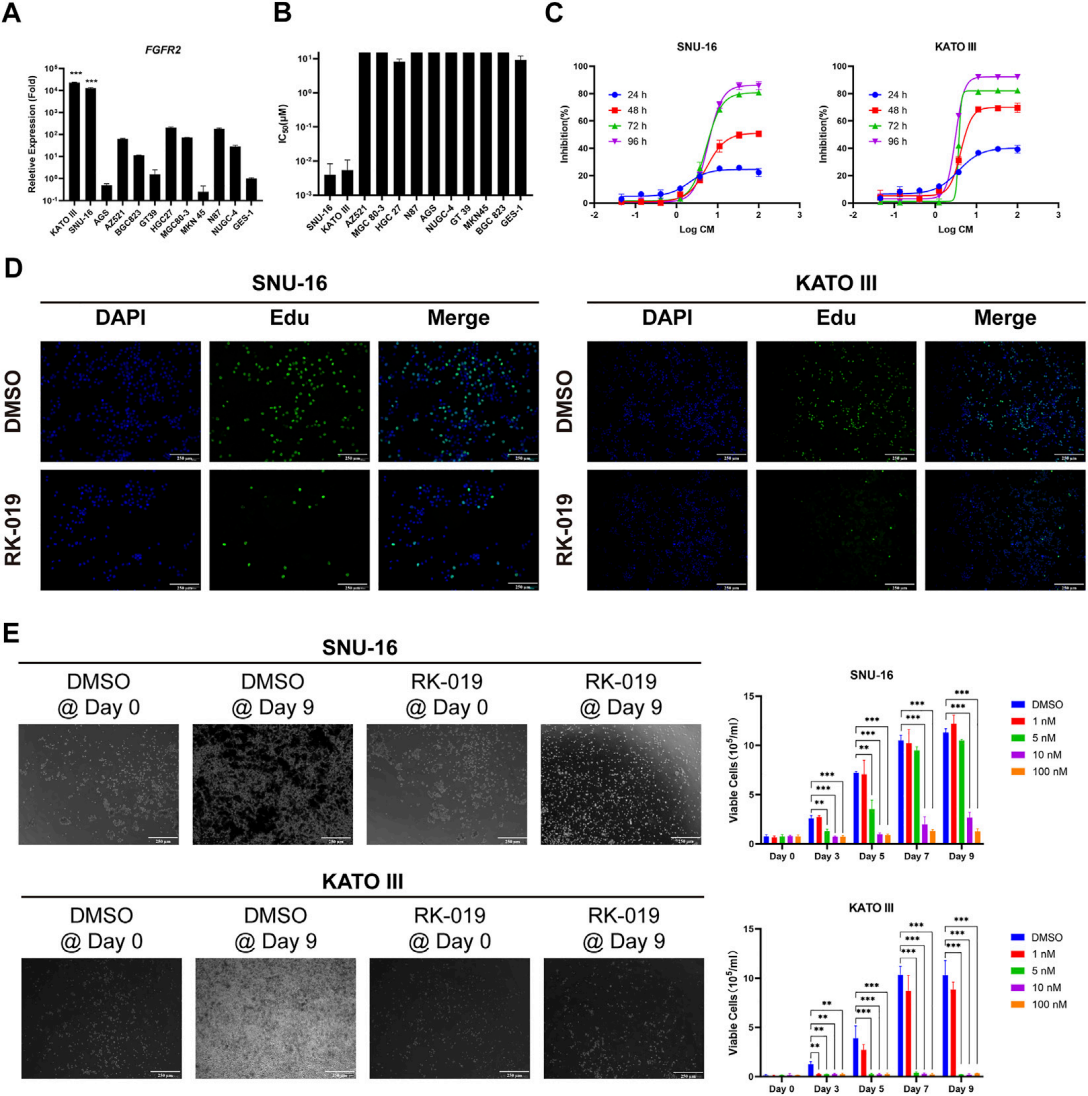
Anti-proliferative action of RK-019. **(A)** qRT-PCR analysis of the *FGFR2* relative expression level on different types of gastric cancer cell lines. GES-1 was used as control. Data were shown in mean ± SEM. **(B)** IC_50_ of RK-019 measured by MTT assay on different types of gastric cancer cell lines. Data were shown in mean ± SD. **(C)** SNU-16 and KATO III cell lines were treated with different concentrations of RK-019 for 24, 48, 72 and 96 h, respectively. The inhibition ratio indexes by MTT assay were the percentage of cells contrast to DMSO-treated group at respect time. Data were shown in mean ± SD. **(D)** Edu staining was used to detect the cell proliferation phenotype with 100 nM RK-019 treatment for 24 h **(E)** SNU-16 and KATO III were treated with RK-019 for 9 days in different concentrations, cells were counted by trypan blue and took pictures every 2 day. The picture only shows the 100 nM RK-019 treatment group on Day 0 and Day 9. Data were shown in mean ± SD. All the data were compiled from three independently repeat experiments. The significance was determined by Student’s t-test, *p < 0.05; **p < 0.01; ***p < 0.001, for the designated treatment group vs. the DMSO group.

Different GC cell lines were used to verify the biological activity of RK-019, including SNU-16, KATO III, AZ 521, MGC 80–3, HGC 27, N87, AGS, NUGC-4, GT 39, MKN 45, BGC823, and gastric epithelial cell line GES-1. The FGFR2 expression level is relevant to poor pathological features and prognostic, and anti-FGFR2 agents could provide potential effectiveness in GC treatment [22, 31, 32]. Firstly, we measured the *FGFRs* mRNA expression levels in these GC cell lines by qRT-PCR. By comparing the expression data, we have found that *FGFR2*-amp cell lines, SNU-16, and KATO III exhibited the highest mRNA expression level of *FGFR2* (Figure 3A, Supplementary Figure S2). Then, MTT assay was performed to test the viability of GC cell lines following treatment with RK-019. As shown in Figure 3B, SNU-16 and KATO III cell lines were demonstrated to be the most sensitive cell lines to RK-019, with the IC_50_ values of SNU-16 and KATO III cell lines were 3.96 ± 4.4 nM and 5.45 ± 5.3 nM, respectively.

Furthermore, SNU-16 and KATO III cells were exposed to graded concentrations of RK-019 for 24, 48, 72, and 96 h to evaluate the dose- and time-dependent relationships. As shown in Figure 3C, RK-019 showed limited suppression effect after 24 h treatment and obvious suppression effect after 48 and 72 h, but did not significantly increase after 96 h. Moreover, Edu staining was used to verify the changes in cell proliferation. After 24 h of RK-019 administration, the number of Edu-positive stained cells significantly decreased in SNU-16 and KATO III (Figure 3D). The results demonstrated that the inhibition of cell proliferation by RK-019 occurred in a time- and dose-dependent manner. In addition, a prolonged drug administration experiment was performed. SNU-16 and KATO III were treated with 1, 5, 10, and 100 nM of RK-019 for 9 days. Live cell numbers were determined by trypan blue staining and photos were taken every 2 days to verify the RK-019 anti-proliferation activity. As shown in Figure 3E, SNU-16 cells were inhibited by treatment with 10 and 100 nM of RK-019, and KATO III cells was suppressed by treatment with 5, 10, and 100 nM of RK-019.

### RK-019 inhibited FGFR2 phosphorylation and the downstream signal pathway in SNU-16 and KATO III cells

When FGFR is bound to its ligand, auto-phosphorylation at T653/T654 site occurs, which is important for the FGFR kinase activity (Zou et al., 2012). Activated FGFR can phosphorylate downstream proteins, such as FRS2 and PLCγ (Xu et al., 1998; Goetz and Mohammadi, 2013), and further activate PI3K-AKT-mTOR and Ras-Raf-Erk pathways (Cailliau et al., 2001; Kamata et al., 2002; Tsang and Dawid, 2004). FGFR signaling activation could induce cell proliferation and survival through the signaling cascade. Thus, western blot analysis was used to study the molecular mechanisms underlying the anti-tumor effects of RK-019 and phosphorylation levels of FGFR2, and downstream proteins were determined and analyzed in SNU-16 and KATO III cells.

**FIGURE 4:**
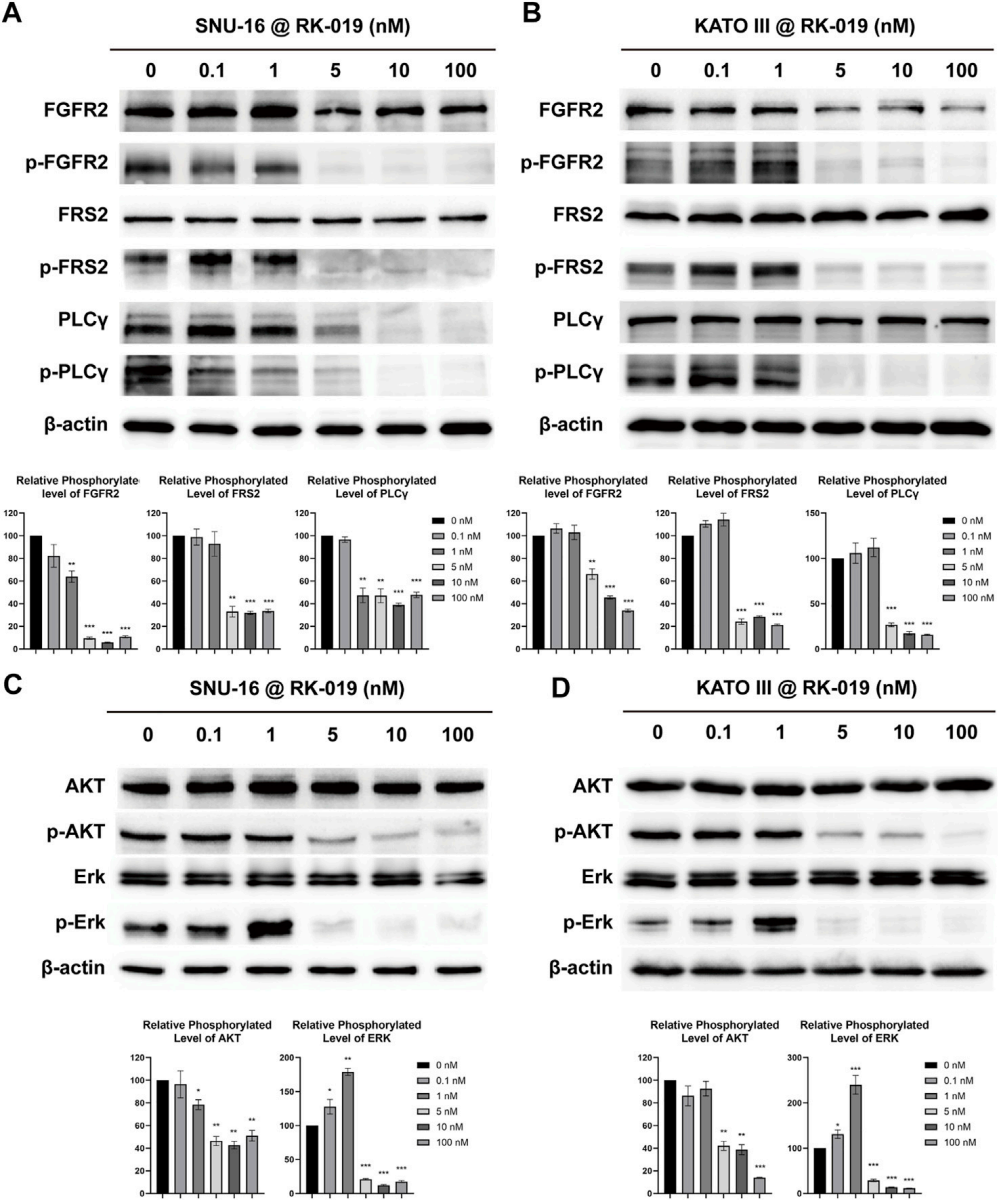
RK-019 effectively inhibits the phosphorylation of FGFR2 and the downstream protein in SNU-16 and KATO III cells. SNU-16 and KATO III cells were treated with different concentrations of RK-019 for 24 h. Then, the cells were harvested and lysed with RIPA, then western blot analyses were performed. Western blot results of FGFR signal related proteins in SNU-16 **(A)** and KATO III **(B)**. Western blot results of downstream of FGFR signal in SNU-16 **(C)** and KATO III **(D)**. Each experiment was repeated for three times independently. ImageJ software was used to quantify the analysis of image profile. Quantification data are performed in mean value ±SD. The significance was determined by Student′s t-test, *p < 0.05; **p < 0.01; ***p < 0.001, for the designated treatment group vs. the DMSO group.

As shown in Figures 4A and B, after treated with 0.1, 1, 5, 10, and 100 nM of RK-019 for 24 h in SNU-16 and KATO III cells, the protein expression level of phosphorylated FGFR2^T653/T654^ was decreased. Furthermore, the FRS2 and PLCγ phosphorylation levels were also significantly decreased, indicating that RK-019 could suppress the FGFR signaling activity by inhibiting its auto-phosphorylation ability. In addition, we observed that the AKT and Erk phosphorylation levels were also decreased (Figures 4C and D). The AKT and Erk phosphorylation levels reflect the activities of the PI3K-AKT-mTOR and Ras-Raf-Erk pathways, which play an important role in cell proliferation and survival (Lawlor and Alessi, 2001; Zhang and Liu, 2002). Overall, our results suggested that RK-019 could inhibit the auto-phosphorylation of FGFR2, affect the activities of downstream proteins, such as FRS2 and PLCγ, and deactivate the PI3K-AKT-mTOR and Ras-Raf-Erk pathways, resulting in cell proliferation inhibition.

### RK-019 induced G0/G1 phase arrest

**FIGURE 5:**
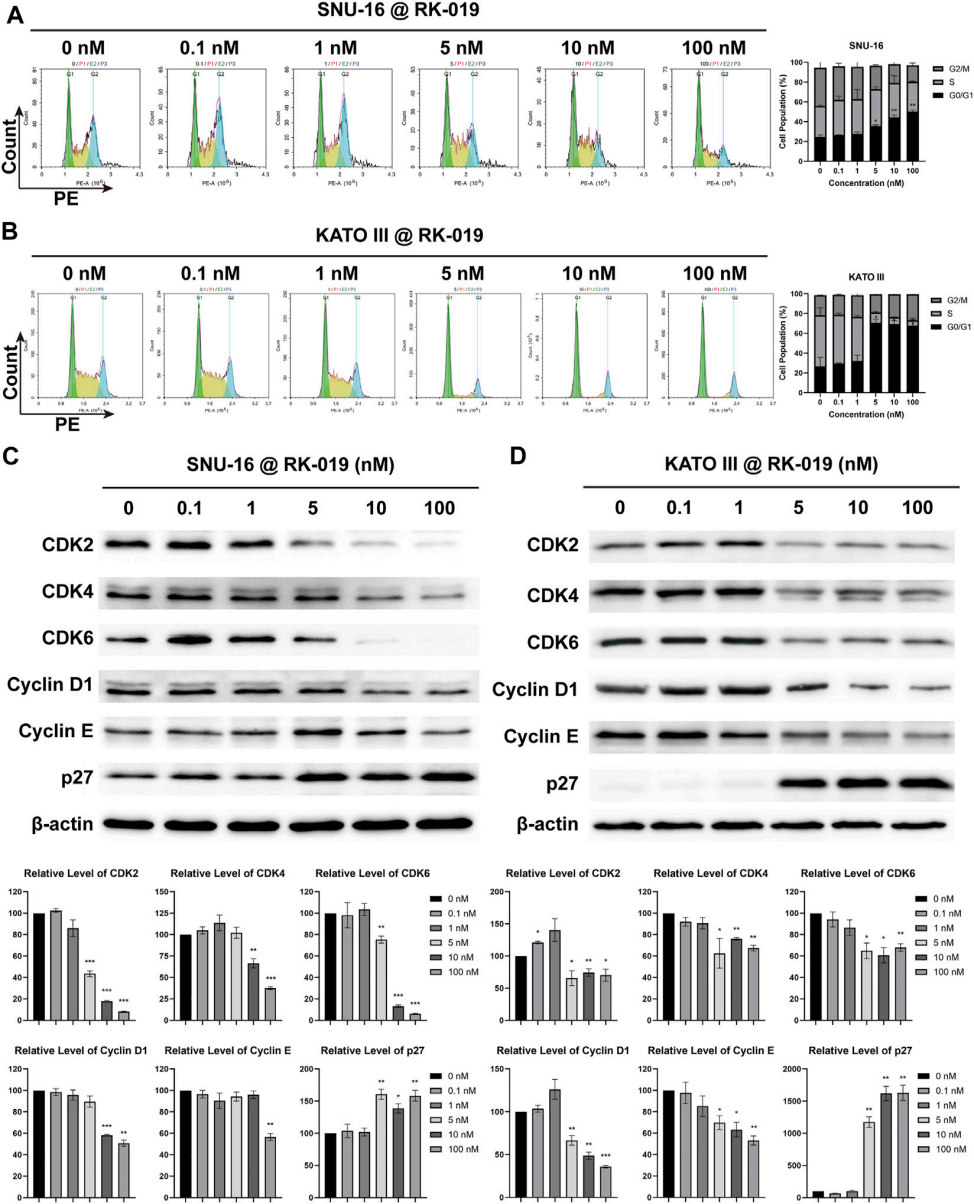
RK-019 induced G0/G1 phase arrest. SNU-16 and KATO III cells were treated with different concentrations of RK-019 for 24 h **(A)**, **(B)** FCM analysis cell cycle distribution by PI staining. Cell cycle distribution was analyzed and quantified by the NovoExpress software. Values were shown by mean ± SD. **(C)**, **(D)** Western blot of cell cycle related proteins and quantification analysis. ImageJ software was used to quantify the analysis of image profile. Quantification data were performed in mean value ±SD. Each experiment was repeated for three times independently. Significance was determined by Student’s t-test *p < 0.05; **p < 0.01; ***p < 0.001, for the designated treatment group vs. the DMSO group.

The FCM analysis was performed to detect the cell cycle distribution. As shown in Figure 5A and B, RK-019 induced G0/G1 arrest after 24 h treatment of RK-019 in SNU-16 and KATO III cells. The G0/G1 proportion was raised from 24.75% to 26.81%–50.29% and 67.88% in SNU-16 and KATO III cells, respectively, after 100 nM RK-019 administration.

We also examined the cell cycle related protein expression levels in cells by western blot analysis. As shown in Figures 5C and D, the G0/G1 cell cycle protein expression levels, including CDK2, 4, and 6, and Cyclin D1 and E, were obviously decreased in KATO III and SNU-16 cells, respectively. Meanwhile, the protein expression level of cell cycle suppression factor p27 was both increased in SNU-16 and KATO III cells. The protein expression levels of CDK2, 4, 6, and Cyclin D1 and E are related with G0/G1 arrest, and p27 can mediate the inhibition of cyclin-CDK2 complex function and result in cell cycle arrest (Massagué, 2004). Therefore, we conclude that RK-019 might induce G0/G1 arrest by inhibited the expression levels of cyclin-CDK complex.

### RK-019 induced apoptosis

**FIGURE 6:**
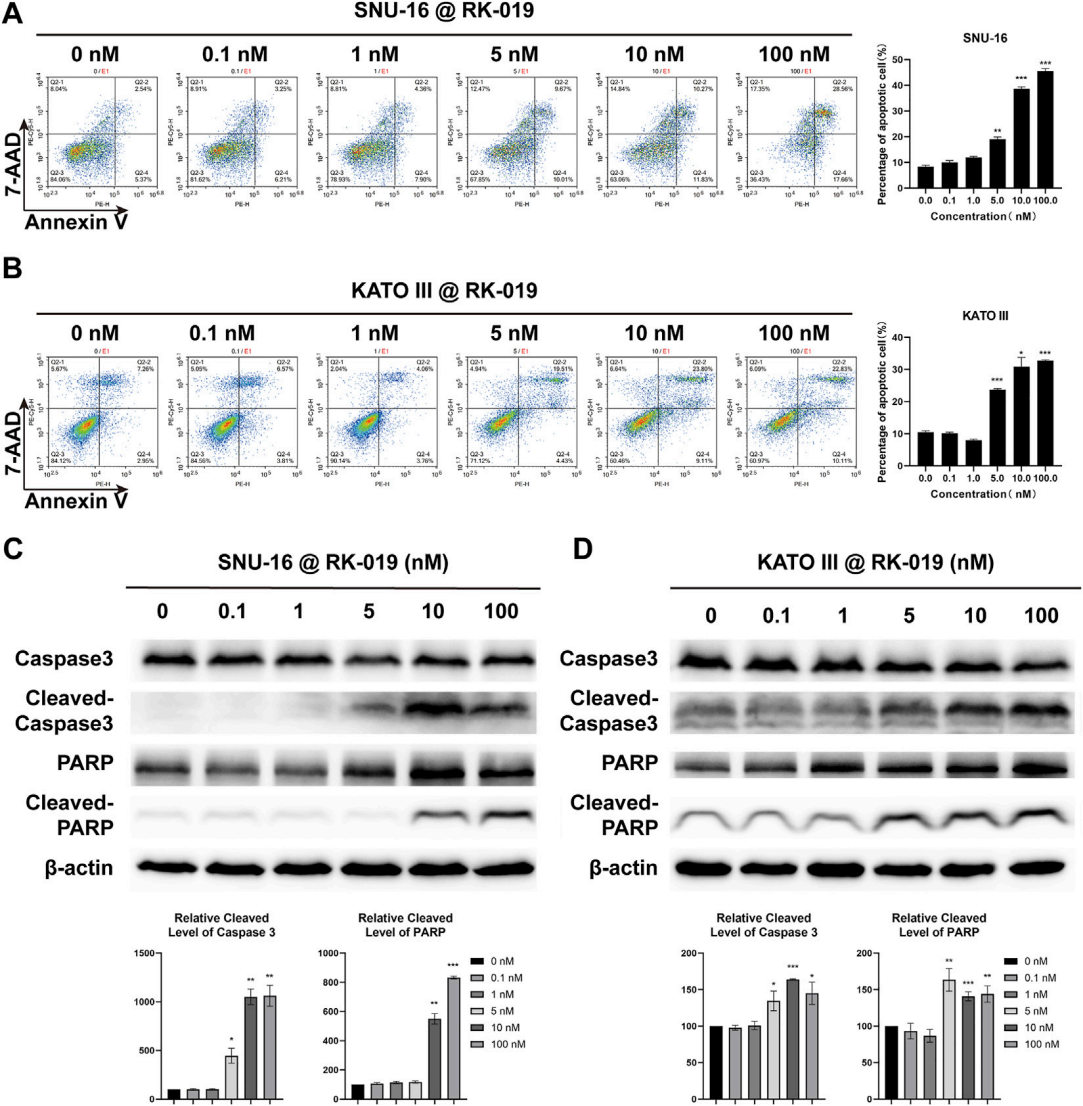
RK-019 induced cell apoptosis. SNU-16 and KATO III cells were treated with different concentrations of RK-019 for 72 h **(A)**, **(B)** FCM analysis cell apoptosis by 7-AAD and Annexin V-PE duel staining and quantified FCM apoptosis analysis data. Values were shown the total number of Q2-2 (early apoptotic) and Q2-4 (late apoptotic) by mean ± SD. **(C)**, **(D)** Western blot of apoptosis related proteins and quantification analysis, data were shown by mean ± SD. Each experiment was repeated for three times independently. Significance was determined by Student’s t-test *p < 0.05; **p < 0.01; ***p < 0.001, for the designated treatment group vs. the DMSO group.

FGFR signal activation in different types of cancer can lead to the apoptosis resistance (Acevedo et al., 2009). Here, we used the Annexin V-PE/7-AAD dual-labeling method to evaluate the apoptosis levels. SNU-16 and KATO III cells were treated with different concentrations of RK-019 for 72 h and analyzed by FCM. The population of apoptotic cells (early and late apoptotic cells) was increased significantly in SNU-16 (45.5%) and KATO III (32.7%) cells (Figures 6A and B), indicating that apoptosis was induced by RK-019. Further, western blot analysis showed that the amount of cleaved caspase-3 in SNU-16 and KATO III was significantly increased after administering RK-019. In addition, the presence of activated caspase-3 and the cleaved version of poly (ADP-ribose) polymerase (PARP) verified the induction of apoptosis (Figures 6C,D).

### RK-019 inhibits cell migration and invasion

**FIGURE 7:**
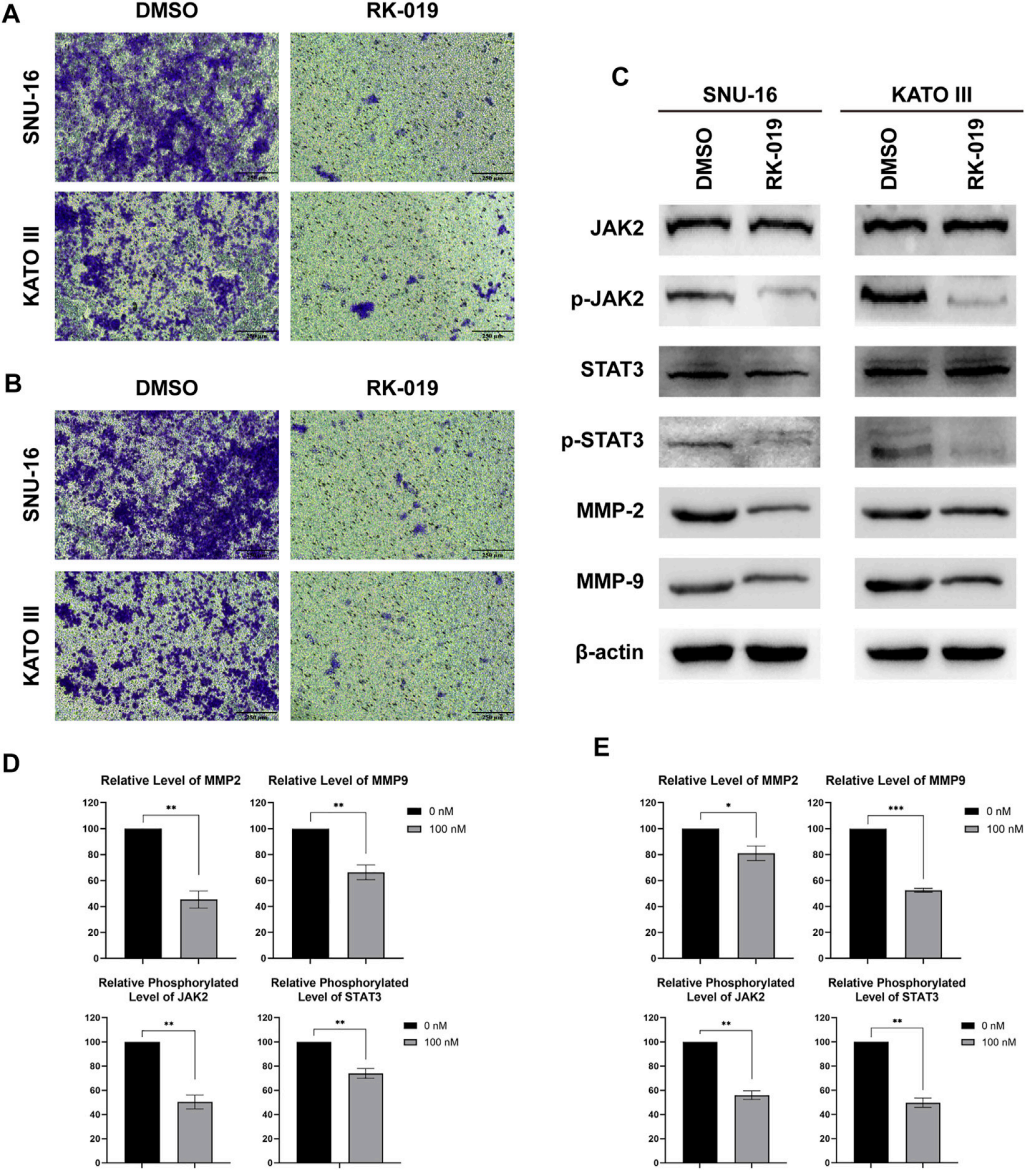
RK-019 inhibit SNU-16 and KATO III migration and invasion. Migration and invasion assay, cells (1 × 10^5^) were seeded in the top chamber of transwell with serum-free medium and treated with RK-019 100 nM for 24 h, then the cells were stained with crystal violet. **(A)** Photographs of migration assay. **(B)** Photographs of invasion assay. **(C)** Western blot of FGFR downstream protein, including JAK2, p-JAK2, STAT3 and p-STAT3, and migration and invasion proteins, MMP-2 and MMP-9. **(D)**, **(E)** Quantification analysis by the ImageJ software of western blot results, data were performed by mean ± SD. Each experiment was repeated for three times independently. Significance was determined by Student’s t-test *p < 0.05; **p < 0.01; ***p < 0.001, for the designated treatment group vs. the DMSO group.

Peritoneal metastasis is one of the leading causes of death in patients with GC (Yao et al., 2020) and FGFRs can phosphorylate STAT to promote cancer cell metastasis (Babina and Turner, 2017). As shown in transwell assays, RK-019 could significantly suppress the migration and invasion ability in SNU-16 and KATO III cells (Figures 7A,B). Furthermore, western blot analysis for migration and invasion-related protein confirmed the effect of RK-019 (Figure 7C). The proteins expression levels of MMP-2 and MMP-9 were reduced obviously after RK-019 treatment. The phosphorylation levels of JAK2 and STAT3, which are FGFRs-related downstream proteins, were decreased. As the STAT signaling pathway can regulate the expression levels of MMP-2 and MMP-9 directly (Xie et al., 2004; Jia et al., 2017), we speculated that RK-019 may suppress the migration and invasion of GC cells by blocking the JAK2-STAT signal axis.

### *In vivo* efficacy of RK-019

Pharmacokinetic profile of RK-019 was conducted in Sprague-Dawley rats (*i.v.*,3 mg/kg; oral, 30 mg/kg). The pharmacokinetic parameters are listed in Table 1. After a single oral administration, RK-019 showed slow absorption (T_max_ = 2.67), the peak plasma concentration (C_max_) was 234.65 ng/ml, the area under the plasma concentration time curve (AUC) was 1,448.41 ng h/mL, the biological half-life (T_1/2_) was 2.83 h, and the oral relative bioavailability was 19.00%.

**TABLE 1:** Pharmacokinetic profiles of RK-019 in rats.

	Dose (mg/kg)	C_max_ (ng/ml)	T_max_ (h)	AUC_0-t_ (ng·h/ml)	MRT (h)	T1/2 (h)	F%
RK-019	3 mg/kg i.v	461.40 ± 47.84	-	762.51 ± 50.79	1.64 ± 0.19	1.58 ± 0.29	-
	30 mg/kg p.o	234.65 ± 77.76	2.67 ± 2.89	1,448.41 ± 313.4	4.49 ± 1.19	2.83 ± 0.37	19.00

**FIGURE 8:**
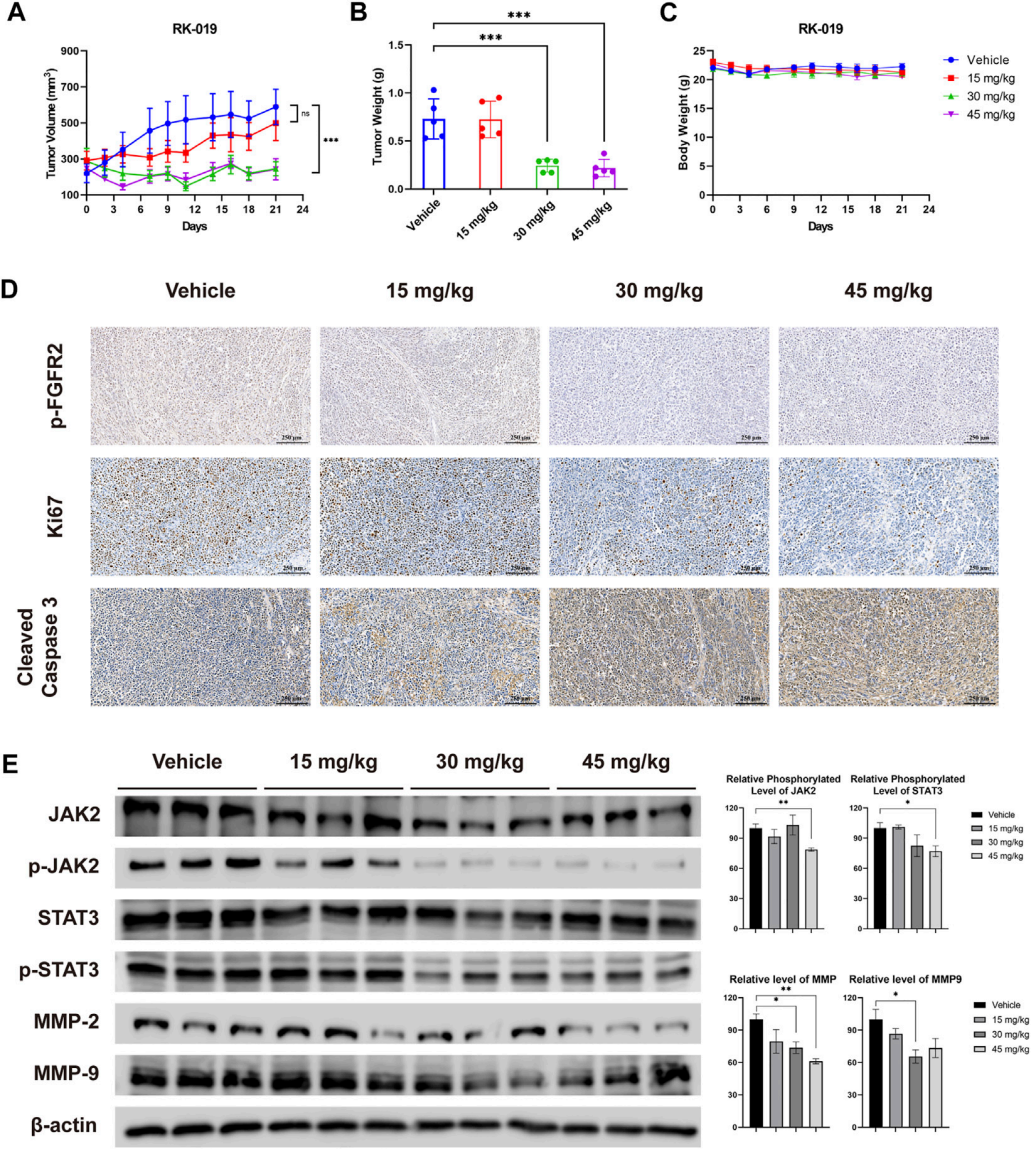
Anti-tumor efficacy of RK-019 in the SNU-16 xenograft model. NOD/SCID mice bearing SNU-16 were orally treated with vehicle or RK-019 once a day for 21 days. Tumor volume and body weight were measured three times *per* week. After 21 days of administration, mice were euthanasia and tumor tissue were collected, and were immunohistochemically analyzed with anti-Ki-67, anti-cleaved caspase-3, and anti p-FGFR2 antibodies. **(A)** Tumor growth curve, data were performed by mean ± SEM. (n = 5) **(B)** Tumor weight, data were performed by mean ± SD. (n = 5) Significance was determined by two-way ANNOVA *p < 0.05; **p < 0.01; ***p < 0.001, for the designated treatment group vs. the Vehicle group. **(C)** Mice body weight curve, data were performed by mean ± SD. (n = 5) **(D)** IHC results of tumor tissue after treatment. **(E)** Western blot analysis of FGFR downstream protein and migration and invasion proteins, including JAK2, p-JAK2, STAT3, p-STAT3, MMP-2, and MMP-9. Each group was randomly selected three tumor tissues, followed by lysis and western blot analysis was performed. Results were quantified by the ImageJ software, and data was performed by mean ± SD. Significance was determined by Student’s t-test *p < 0.05; **p < 0.01; ***p < 0.001, for the designated treatment group vs. the vehicle group.

To investigate the anti-tumor effect of RK-019 *in vivo*, we established a xenograft model using SNU-16 cells. RK-019 was administered at doses of 15, 30, and 45 mg/kg once a day and after 21 days of treatment, the TGI values were 34.3, 85.9, and 83.5%, respectively (Figure 8A). Meanwhile, a significant decrease in tumor weight in the RK-019-treated group was observed compared to that in the vehicle group (Figure 8B).

To validate the results of the *in vivo* assay, we conducted IHC analyses using Ki67 as a cell proliferation marker, cleaved caspase-3 as an apoptotic cell marker, and p-FGFR2 as a FGFR2 signaling activity marker. As shown in Figure 8D, the number of p-FGFR2-positive and Ki67-positive cells decreased, while the number of cleaved-caspase 3-positive cells increased significantly. Moreover, western blot analysis was performed to detecting the influence of RK-019 on JAK2-STAT signal axis *in vivo* (Figure 8D). Although the changes of relative phosphorylation level of JAK2 and STAT3 are not significant, the JAK2 and STAT3 phosphorylation level decreased remarkably in 30 and 45 mg/kg RK-019 treatment groups. The downstream proteins expression of this axis, MMP2 and MMP9, have also decreased after RK-019 treatment. These results suggested that RK-019 could block the FGFR signaling pathway *in vivo*, and result in proliferation arrest and cell apoptosis induction, corroborating the *in vitro* observations. In the meantime, RK-019 exhibit the potential ability, suppress the migration, and invasion of GC cells *in vivo.*

**FIGURE 9:**
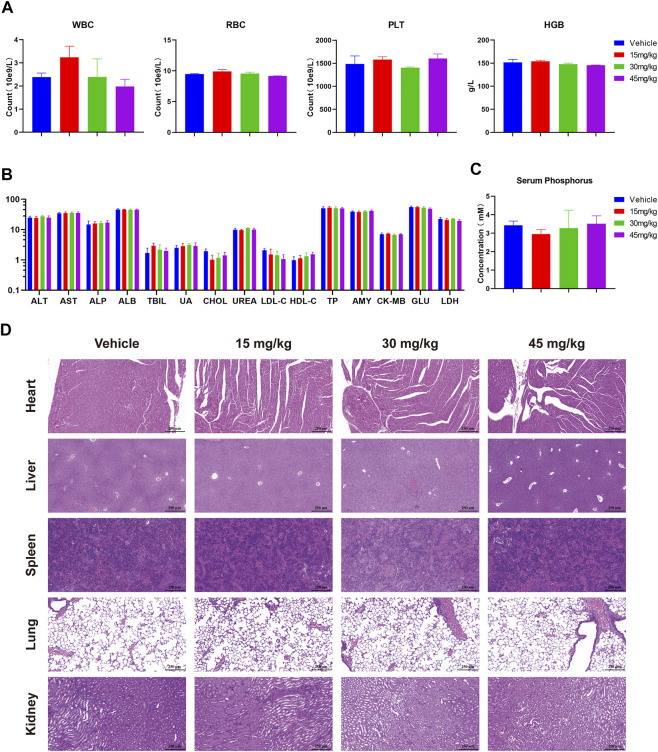
Safety profile of RK-019 in the xenograft mice model. After 21 days of RK-019 treatment, the mice were euthanasia, then blood and organs were extracted and analysis. Values in graphics were plotted as mean ± SD (n = 5). **(A)** Complete blood count data. **(B)** Blood biochemical analysis. **(C)** Serum phosphorus analysis. **(D)** H&E staining of pathological section in the heart, liver, spleen, lungs, and kidneys.

Meanwhile, preliminary toxicity of RK-019 was evaluated. During the treatment, no significant body weight change occurred (Figure 8C). Moreover, no pathological changes were observed in the harvested organs of the RK-019-treated group (Figure 9D), and hematopoietic toxicity was absent (Figures 9A–C) at the end of treatment, suggesting that the mice were tolerant to RK-019 treatment. Taken together, RK-019 inhibited tumor growth in the SNU-16 xenograft model by inhibiting cell proliferation and inducing cell apoptosis.

## Discussion

FGFR signaling is an important regulatory pathway. However, FGFR signaling disorder is closely related to many diseases, such as cancer and fibrosis (Robertson et al., 2000). Abnormally activated FGFR signaling in cancer activates a series of signaling pathways, such as PI3K-AKT-mTOR, Ras-Raf-Erk, and JAK-STAT, which results in uncontrolled cell proliferation, metastasis, and avoidance of cell death (Gschwind et al., 2004). Preclinical studies have identified FGFRs as a potential therapeutic target in many types of cancers, including lung, breast, gastric, and hematologic cancers. Excitingly, FGFR inhibitors, including Erdafitinib (JNJ-42756493) and Infigratinib (BGJ-398), have been approved for urothelial carcinoma and cholangiocarcinoma treatment (Markham, 2019; Montazeri and Bellmunt, 2020; Botrus et al., 2021).

GC is a highly heterogeneous disease from morphological and molecular standpoints (Gullo et al., 2018). This results in heterogeneity of treatment effect and makes the GC treatment development more difficult than other types of cancers. Until now, therapeutic schedules for GC are very limited. Traditional chemotherapy drugs, such as anti-tumor platinum drugs, docetaxel, and 5-Fu, still are the most common drugs in gastric cancer treatment (Smyth et al., 2020). Compared to the chemotherapy, targeted therapy exhibited more advantages in efficacy and safety. However, targeted drugs for GC treatment are very limited. Studies have revealed FGFR2 as a potential target for GC treatment (Lengyel et al., 2022). Therefore, discovery targeted therapy for FGFR2 is very significant.

However, FGFRs inhibitors still are face many challenges. Firstly, although regulation of FGFR signaling cascades has been widely investigated, their unique function and drug resistance mechanisms remain unclear (Babina and Turner, 2017; Simons, 2021). Use of FGFRs inhibitors might cause a series of problems such as mutations conferring resistance to FGFR-targeting drugs and side-effects (Yue et al., 2021). On the other hand, due to the patient heterogeneity, FGFRs kinase inhibitors and anti-body drug conjugates had shown lower efficacy than expected, and drug toxicity, multiple GC treatment clinical trials targeted on FGFRs have not made any progress (Repetto et al., 2021; Lengyel et al., 2022). Hence, the discovery of novel FGFRs inhibitors is necessary.

In our present study, we demonstrated a novel small molecule RK-019 by screening our library of pharmacologically active compounds. In the following research, we found RK-019 exhibited great inhibitory capacity and selectivity against the FGFRs family kinases, the IC_50_ were 9.1 nM (FGFR1), 4.6 nM (FGFR2), 26.3 nM (FGFR3), and 40.7 nM (FGFR4), respectively. This indicated RK-019 is a pan-FGFR inhibitor with great kinase inhibitory activity.

Then, we tested the anti-proliferative effect of RK-019 on multiple GC cell lines and found that it could efficiently inhibit the proliferation of *FGFR2*-amp GC cell lines, SNU-16 and KATO III, with average IC_50_ values of 3.96 ± 4.4 nM and 5.45 ± 5.3 nM, respectively. These IC_50_ values were significantly different from that of the gastric epithelial cells GES-1 or other types of GC cell lines, indicating that RK-019 could inhibit *FGFR2*-amp GC cells. However, prolonged treatment of SNU-16 cells with RK-019 (5 and 10 nM) showed contrasting results, likely due to the activation of drug resistance pathways. Thus, in the future, we will focus on identifying the mechanism of drug resistance and developing anti-drug resistance strategies to enhance the anti-tumor effect.

Our results verified that RK-019 could inhibit FGFR2^T653/T654^ auto-phosphorylation, which is the critical modification for the FGFR2 kinase activity and could prevent the phosphorylation of downstream proteins, including FRS2 and PLCγ. Phosphorylated FRS2 and PLCγ play a central role in the FGFR pathway activity, triggering PI3K-AKT-mTOR and Ras-Raf-Erk pathways (Katoh and Katoh, 2006; Acevedo et al., 2009; Ahmad et al., 2012; Ornitz and Itoh, 2015). In our study, we observed a decrease in the phosphorylation of AKT and Erk, which are known regulators of cell proliferation and survival. Furthermore, FCM and western blot results revealed G0/G1 phase arrest, validating our speculation about the inhibitory effects on the FGFR signaling.

Interestingly, although cell cycle arrest occurred immediately after RK-019 treatment, apoptosis was delayed. Apoptosis occurred after 72 h treatment, indicating that apoptosis might not be directly triggered by the inhibition of FGFR signaling. In addition, we found that apoptosis induced by RK-019 was not mediated by mitochondria. Mitochondrial membrane potential was increased, while the expression levels of mitochondrial apoptosis-related proteins did not change as expected after RK-019 treatment (Data not shown). Thus, we presume that RK-019 induced apoptosis *via* non-classic pathways.

Furthermore, we found that RK-019 could inhibit cell migration and invasion in SNU-16 and KATO III cells. Peritoneal metastasis is a leading cause of death in patients with gastric carcinoma. However, there are limited treatment options and no targeted therapy or immunotherapy for gastric carcinoma (Yao et al., 2020). RK-019 could inhibit the FGFR2 activity and repress JAK2 phosphorylation, thus mediating the STAT3 activity and decreasing the MMP-2 and MMP-9 levels. These findings demonstrate the ability of RK-019 to control cancer cell metastasis, which is crucial in GC therapy.

Finally, we verified the anti-tumor activity *in vivo* using the SNU-16 xenograft model. The results showed that RK-019 (30 mg/kg, daily oral administration for 21 days) could remarkably suppress SNU-16 tumor growth, with an inhibitory ratio of 85.9%. Furthermore, IHC revealed that RK-019 could prevent FGFR2 phosphorylation, induce apoptosis, and inhibit cell proliferation in the tumor sections.

Taken together, we have reported here a novel pan-FGFR inhibitor, RK-019, which exhibited excellent anti-tumorigenic activity against *FGFR2*-amp GC *in vitro* and *in vivo*.

## Data Availability

The original contributions presented in the study are included in the article/Supplementary Material; further inquiries can be directed to the corresponding authors.

## Glossary

GC: Gastric cancer
FGFR: Fibroblast growth factor receptor
FGF: Fibroblast growth factor
EMT: Epithelial-to-mesenchymal transition
MTT: 3-(4,5-Dimethyl-2-thiazolyl)-2,5-diphenyl-2-H-tetra-zolium bromide
DMSO: Dimethyl sulfoxide
qRT-PCR: Quantitative reverse transcription PCR
FCM: Flow cytometry: Flow cytometry
PBS: Phosphate buffered solution
SDS-PAGE: Sodium dodecyl sulphate-polyacrylamide gel electrophoresis
PVDF: Polyvinylidene difluoride
SPF: Specific-pathogen free
LC-MS/MS: Liquid chromatography–mass spectrometry/mass spectrometry
PK: Pharmacokinetics
FCM: Flow cytometry: Flow cytometry
PI: Propidium iodide
PARP: Poly (ADP-ribose) polymerase
CBC: Complete blood count
TGI: Tumor growth inhibition
IC50: Median-inhibitory concentration
ATP: Adenosine triphosphate
AUC: Area under the plasma concentration time curve
IHC: Immunohistochemistry
FRS2: Fibroblast growth factor receptor substrate 2
PLCγ: Phosphoinositide phospholipase C gamma
Erk: Extracellular signal-regulated kinase
JAK: Janus kinase
STAT: Signal transducer and activator of transcription
CDK: Cyclin-dependent kinases
MMP: Matrix metalloproteinases
PI3K: Phosphatidylinositol 3 kinase.
