# Supplementary material for: A novel small molecule RK-019 inhibits FGFR2-amplification gastric cancer cell proliferation and induces apoptosis in vitro and in vivo
Source: Front Pharmacol. 2022 Sep 21;13:998199. doi: 10.3389/fphar.2022.998199 (PMC9532703; doi:10.3389/fphar.2022.998199)
Supplement: Supplementary file 1 [file Table1.DOCX]

**Supplementary Table: The inhibition ratio of RK-019 on 422 kinds of kinases at 1 μM**

| **Kinase** | **Inhibition（%）**  **@ 1 µM RK-019** | **Kinase** | **Inhibition（%）**  **@ 1 µM RK-019** |
| --- | --- | --- | --- |
| AAK1(h) | 91 | CaMKIIβ(h) | 80 |
| Abl(h) | 40 | CaMKIIγ(h) | 106 |
| Abl(m) | 17 | CaMKIδ(h) | 98 |
| Abl (H396P) (h) | 49 | CaMKIIδ(h) | 108 |
| Abl (M351T)(h) | 34 | CaMKIV(h) | 113 |
| Abl (Q252H) (h) | 46 | CaMKK1(h) | 105 |
| Abl(T315I)(h) | 66 | CaMKK2(h) | 95 |
| Abl(Y253F)(h) | 54 | Cdc7/cyclinB1(h) | 87 |
| ACK1(h) | 104 | CDK1/cyclinB(h) | 99 |
| ACTR2(h) | 85 | CDK2/cyclinA(h) | 115 |
| ALK(h) | 85 | CDK2/cyclinE(h) | 123 |
| ALK1(h) | 98 | CDK3/cyclinE(h) | 90 |
| ALK2(h) | 90 | CDK4/cyclinD3(h) | 101 |
| ALK4(h) | 91 | CDK5/p25(h) | 112 |
| ALK6(h) | 107 | CDK5/p35(h) | 106 |
| Arg(h) | 86 | CDK6/cyclinD3(h) | 106 |
| AMPKα1(h) | 107 | CDK7/cyclinH/MAT1(h) | 108 |
| AMPKα2(h) | 109 | CDK9/cyclin T1(h) | 99 |
| A-Raf(h) | 133 | CDK12/cyclinK(h) | 109 |
| Arg(m) | 88 | CDK13/cyclinK(h) | 109 |
| ARK5(h) | 146 | CDK14/cyclinY(h) | 91 |
| ASK1(h) | 118 | CDK16/cyclinY(h) | 108 |
| Aurora-A(h) | 133 | CDK17/cyclinY(h) | 104 |
| Aurora-B(h) | 87 | CDK18/cyclinY(h) | 96 |
| Aurora-C(h) | 86 | CDKL1(h) | 97 |
| Axl(h) | 116 | CDKL2(h) | 110 |
| BIKe(h) | 93 | CDKL3(h) | 102 |
| Blk(h) | 70 | CDKL4(h) | 90 |
| Blk(m) | 53 | ChaK1(h) | 100 |
| BMPR2(h) | 116 | CHK1(h) | 90 |
| Bmx(h) | 64 | CHK2(h) | 96 |
| BRK(h) | 89 | CHK2(I157T)(h) | 100 |
| BrSK1(h) | 106 | CHK2(R145W)(h) | 100 |
| BrSK2(h) | 96 | CK1ε(h) | 99 |
| BTK(h) | 72 | CK1γ1(h) | 92 |
| BTK(R28H)(h) | 83 | CK1γ2(h) | 97 |
| B-Raf(h) | 124 | CK1γ3(h) | 87 |
| B-Raf(V599E)(h) | 70 | CK1δ(h) | 94 |
| CaMKI(h) | 101 | CK1(y) | 94 |
| CaMKIß(h) | 97 | CK2(h) | 112 |
| CaMKIγ(h) | 93 | CK2α1(h) | 95 |
| CaMKIIα(h) | 64 | CK2α2(h) | 105 |
| CLIK1(h) | 102 | EphB3(h) | 89 |
| CLK1(h) | 104 | EphB4(h) | 109 |
| CLK2(h) | 107 | ErbB2(h) | 94 |
| CLK3(h) | 102 | ErbB4(h) | 105 |
| CLK4(h) | 89 | FAK(h) | 105 |
| cKit(h) | 73 | Fer(h) | 127 |
| cKit(D816V)(h) | 93 | Fes(h) | 106 |
| cKit(D816H)(h) | 58 | FGFR1(h) | -1 |
| cKit(V560G)(h) | 14 | FGFR1(V561M)(h) | 70 |
| cKit(V654A)(h) | 104 | FGFR2(h) | 1 |
| CRIK(h) | 117 | FGFR2(N549H)(h) | -1 |
| CSK(h) | 94 | FGFR3(h) | -3 |
| c-RAF(h) | 94 | FGFR4(h) | 5 |
| cSRC(h) | 54 | Fgr(h) | 54 |
| DAPK1(h) | 100 | Flt1(h) | 29 |
| DAPK2(h) | 97 | Flt3(D835Y)(h) | 86 |
| DCAMKL2(h) | 126 | Flt3(h) | 123 |
| DCAMKL3(h) | 127 | Flt4(h) | 3 |
| DDR1(h) | 19 | Fms(h) | 16 |
| DDR2(h) | 109 | Fms(Y969C)(h) | 31 |
| DMPK(h) | 98 | Fyn(h) | 62 |
| DRAK1(h) | 95 | GCK(h) | 98 |
| DRAK2(h) | 96 | GCN2(h) | 134 |
| DYRK1A(h) | 101 | GRK1(h) | 94 |
| DYRK1B(h) | 97 | GRK2(h) | 103 |
| DYRK2(h) | 94 | GRK3(h) | 123 |
| DYRK3(h) | 88 | GRK5(h) | 135 |
| eEF-2K(h) | 97 | GRK6(h) | 114 |
| EGFR(h) | 119 | GRK7(h) | 108 |
| EGFR(L858R)(h) | 114 | GSK3α(h) | 85 |
| EGFR(L861Q)(h) | 103 | GSK3β(h) | 96 |
| EGFR(T790M)(h) | 90 | Haspin(h) | 105 |
| EGFR(T790M,L858R)(h) | 92 | Hck(h) | 27 |
| EphA1(h) | 19 | Hck(h) activated | 60 |
| EphA2(h) | 61 | HIPK1(h) | 93 |
| EphA3(h) | 60 | HIPK2(h) | 83 |
| EphA4(h) | 101 | HIPK3(h) | 94 |
| EphA5(h) | 45 | HIPK4(h) | 100 |
| EphA7(h) | 108 | HPK1(h) | 95 |
| EphA8(h) | 59 | HRI(h) | 100 |
| EphB2(h) | 96 | ICK(h) | 119 |
| EphB1(h) | 58 | IGF-1R(h) | 104 |
| IGF-1R(h), activated | 101 | MEKK2(h) | 96 |
| IKKα(h) | 100 | MEKK3(h) | 103 |
| IKKβ(h) | 90 | MELK(h) | 104 |
| IKKε(h) | 107 | Mer(h) | 108 |
| IR(h) | 118 | Met(h) | 97 |
| IR(h), activated | 95 | Met(D1246H)(h) | 89 |
| IRE1(h) | 93 | Met(D1246N)(h) | 89 |
| IRR(h) | 104 | Met(M1268T)(h) | 71 |
| IRAK1(h) | 103 | Met(Y1248C)(h) | 85 |
| IRAK4(h) | 100 | Met(Y1248D)(h) | 103 |
| Itk(h) | 104 | Met(Y1248H)(h) | 113 |
| JAK1(h) | 120 | MINK(h) | 133 |
| JAK2(h) | 106 | MKK3(h) | 96 |
| JAK3(h) | 97 | MKK4(m) | 107 |
| JNK1α1(h) | 96 | MKK6(h) | 83 |
| JNK2α2(h) | 99 | MLCK(h) | 92 |
| JNK3(h) | 91 | MLK1(h) | 99 |
| KDR(h) | 9 | MLK2(h) | 103 |
| Lck(h) | 30 | MLK3(h) | 98 |
| Lck(h) activated | 36 | Mnk2(h) | 108 |
| LIMK1(h) | 103 | MOK(h) | 97 |
| LIMK2(h) | 98 | MRCKα(h) | 103 |
| LKB1(h) | 94 | MRCKβ(h) | 108 |
| LOK(h) | 76 | MRCKγ(h) | 94 |
| Lyn(h) | 6 | MSK1(h) | 95 |
| Lyn(m) | 23 | MSK2(h) | 101 |
| LRRK2(h) | 80 | MSSK1(h) | 135 |
| LTK(h) | 101 | MST1(h) | 121 |
| MAK(h) | 110 | MST2(h) | 97 |
| MAPK1(h) | 116 | MST3(h) | 122 |
| MAPK2(h) | 115 | MST4(h) | 90 |
| MAPK2(m) | 109 | mTOR(h) | 105 |
| MAP4K3(h) | 132 | mTOR/FKBP12(h) | 92 |
| MAP4K4(h) | 82 | MuSK(h) | 105 |
| MAP4K5(h) | 103 | MYLK2(h) | 88 |
| MAPKAP-K2(h) | 121 | MYO3B(h) | 106 |
| MAPKAP-K3(h) | 109 | NDR2(h) | 108 |
| MEK1(h) | 79 | NEK1(h) | 52 |
| MEK2(h) | 85 | NEK2(h) | 80 |
| MARK1(h) | 93 | NEK4(h) | 109 |
| MARK3(h) | 80 | NEK3(h) | 87 |
| MARK4(h) | 106 | NEK6(h) | 97 |
| NEK7(h) | 99 | PKCθ(h) | 119 |
| NEK9(h) | 91 | PKCζ(h) | 91 |
| NIM1(h) | 103 | PKD2(h) | 98 |
| NEK11(h) | 118 | PKD3(h) | 99 |
| NLK(h) | 86 | PKG1α(h) | 110 |
| NUAK2(h) | 90 | PKG1β(h) | 103 |
| p70S6K(h) | 119 | PKR(h) | 50 |
| PAK1(h) | 102 | Plk1(h) | 117 |
| PAK2(h) | 98 | Plk3(h) | 101 |
| PAK4(h) | 82 | Plk4(h) | 91 |
| PAK3(h) | 97 | PRAK(h) | 95 |
| PAK5(h) | 101 | PRKG2(h) | 100 |
| PAK6(h) | 96 | PRK1(h) | 105 |
| PAR-1Bα(h) | 92 | PRK2(h) | 106 |
| PASK(h) | 96 | PrKX(h) | 98 |
| PEK(h) | 85 | PRP4(h) | 90 |
| PDGFRα(h) | 50 | PTK5(h) | 107 |
| PDGFRα(D842V)(h) | 22 | Pyk2(h) | 112 |
| PDGFRα(V561D)(h) | 1 | Ret(h) | 5 |
| PDGFRβ(h) | 54 | Ret (V804L)(h) | 92 |
| PDHK2(h) | 104 | Ret(V804M)(h) | 56 |
| PDHK4(h) | 93 | RIPK1(h) | 85 |
| PDK1(h) | 110 | RIPK2(h) | 46 |
| PhKγ1(h) | 93 | ROCK-I(h) | 101 |
| PhKγ2(h) | 88 | ROCK-II(h) | 105 |
| Pim-1(h) | 111 | ROCK-II(r) | 91 |
| Pim-2(h) | 98 | Ron(h) | 124 |
| Pim-3(h) | 114 | Ros(h) | 101 |
| PKA(h) | 94 | Rse(h) | 104 |
| PKAcβ(h) | 98 | Rsk1(h) | 120 |
| PKBα(h) | 102 | Rsk1(r) | 96 |
| PKBβ(h) | 84 | Rsk2(h) | 149 |
| PKBγ(h) | 94 | Rsk3(h) | 100 |
| PKCα(h) | 97 | Rsk4(h) | 146 |
| PKCβI(h) | 102 | SAPK2a(h) | 100 |
| PKCβII(h) | 111 | SAPK2a(T106M)(h) | 90 |
| PKCγ(h) | 92 | SAPK2b(h) | 94 |
| PKCδ(h) | 99 | SAPK3(h) | 99 |
| PKCε(h) | 88 | SAPK4(h) | 112 |
| PKCη(h) | 108 | SBK1(h) | 96 |
| PKCι(h) | 100 | SGK(h) | 104 |
| PKCμ(h) | 107 | SGK2(h) | 79 |
| SGK3(h) | 97 | TTBK1(h) | 90 |
| SIK(h) | 89 | TTBK2(h) | 82 |
| SIK2(h) | 99 | TTK(h) | 98 |
| SIK3(h) | 104 | Txk(h) | 106 |
| SLK(h) | 99 | TYK2(h) | 96 |
| Snk(h) | 109 | ULK1(h) | 105 |
| SNRK(h) | 92 | ULK2(h) | 107 |
| Src(1-530)(h) | 45 | ULK3(h) | 91 |
| Src(T341M)(h) | 98 | VRK1(h) | 98 |
| SRMS(h) | 123 | VRK2(h) | 104 |
| SRPK1(h) | 112 | Wee1(h) | 92 |
| SRPK2(h) | 134 | Wee1B(h) | 107 |
| STK16(h) | 113 | WNK1(h) | 95 |
| STK25(h) | 66 | WNK2(h) | 103 |
| STK32A(h) | 86 | WNK3(h) | 110 |
| STK32B(h) | 123 | WNK4(h) | 86 |
| STK32C(h) | 72 | Yes(h) | 29 |
| STK33(h) | 98 | ZAK(h) | 80 |
| Syk(h) | 116 | ZAP-70(h) | 119 |
| TAF1L(h) | 100 | ZIPK(h) | 96 |
| TAK1(h) | 102 | ATM(h) | 105 |
| TAO1(h) | 115 | ATR/ATRIP(h) | 107 |
| TAO2(h) | 95 | DNA-PK(h) | 101 |
| TAO3(h) | 100 | PI3 Kinase (p110b/p85a)(h) | 100 |
| TBK1(h) | 101 | PI3 Kinase (p120g)(h) | 102 |
| Tec(h) activated | 96 | PI3 Kinase (p110d/p85a)(h) | 93 |
| TGFBR1(h) | 83 | PI3 Kinase (p110a/p85a)(m) | 101 |
| TGFBR2(h) | 85 | PI3 Kinase (p110a/p65a)(m) | 100 |
| Tie2 (h) | 105 | PI3 Kinase (p110a(E545K)/p85a)(m) | 102 |
| Tie2(R849W)(h) | 82 | PI3 Kinase (p110a(H1047R)/p85a)(m) | 100 |
| Tie2(Y897S)(h) | 77 | PI3 Kinase (p110b/p85b)(m) | 99 |
| TLK1(h) | 98 | PI3 Kinase (p110b/p85a)(m) | 98 |
| TLK2(h) | 124 | PI3 Kinase (p110d/p85a)(m) | 98 |
| TNIK(h) | 42 | PI3 Kinase (p110a(E542K)/p85a)(m) | 99 |
| TRB2(h) | 136 | PI3 Kinase (p110a/p85a)(h) | 101 |
| TrkA(h) | 92 | PI3 Kinase (p110a(E542K)/p85a)(h) | 98 |
| TrkB(h) | 94 | PI3 Kinase (p110a(H1047R)/p85a)(h) | 100 |
| TrkC(h) | 86 | PI3 Kinase (p110a(E545K)/p85a)(h) | 100 |
| TSSK1(h) | 109 | PI3 Kinase (p110a/p65a)(h) | 94 |
| TSSK2(h) | 91 | PI3KC2a(h) | 104 |
| TSSK3(h) | 102 | PI3KC2g(h) | 102 |
| TSSK4(h) | 106 | PIP4K2a(h) | 102 |
| PIP5K1a(h) | 103 | PIP5K1g(h) | 97 |
