# Supplementary figures and images for: A novel small molecule RK-019 inhibits FGFR2-amplification gastric cancer cell proliferation and induces apoptosis in vitro and in vivo
Source: Front Pharmacol. 2022 Sep 21;13:998199. doi: 10.3389/fphar.2022.998199 (PMC9532703; doi:10.3389/fphar.2022.998199)

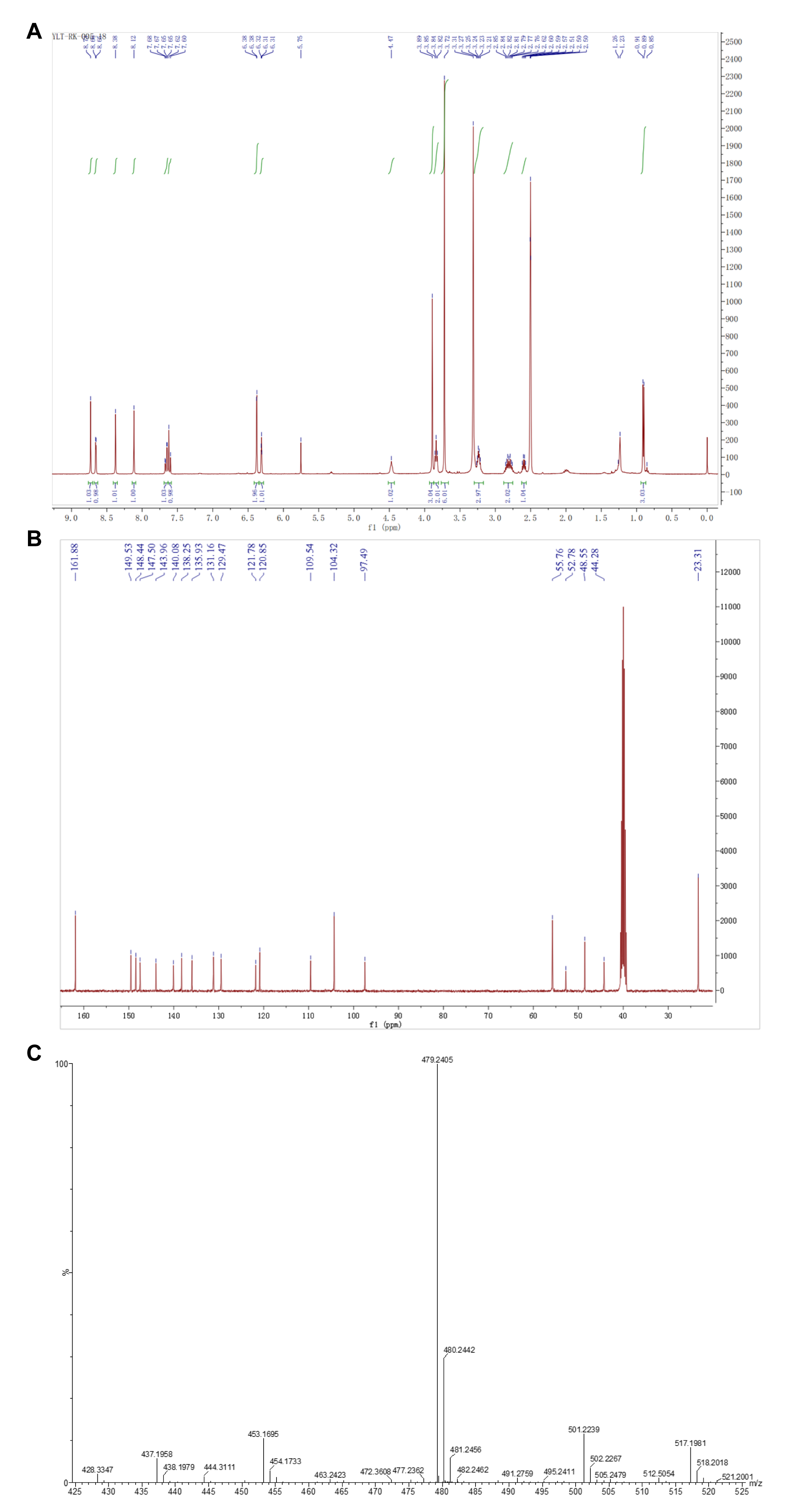

Supplement: Supplementary file 2 [file Image3.TIF]

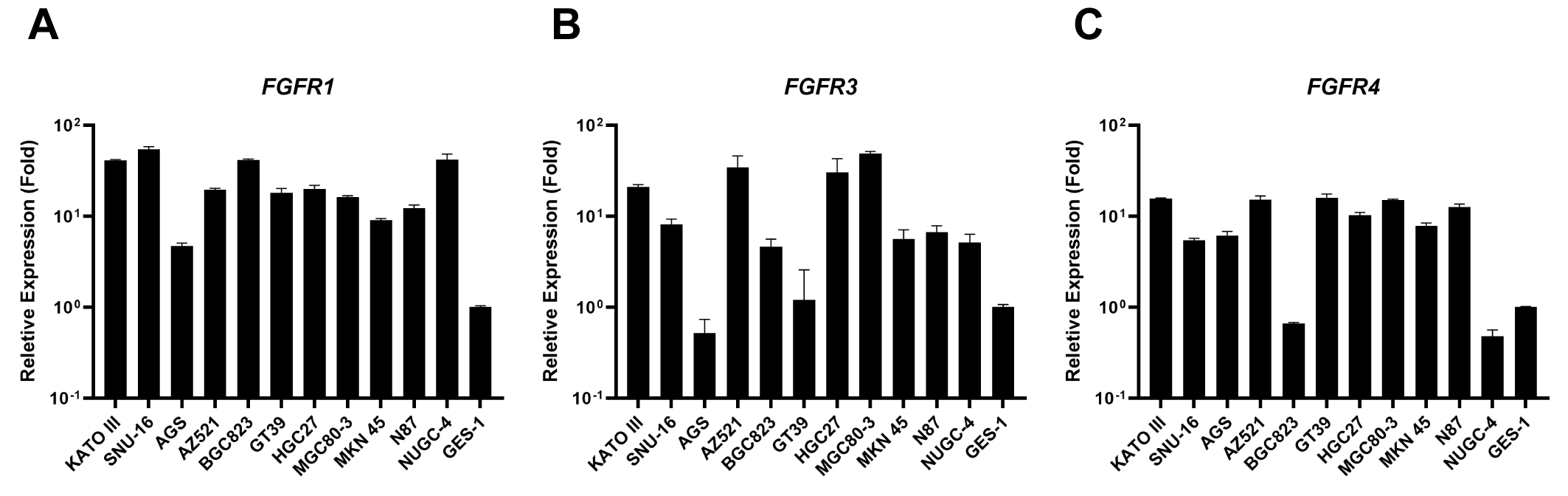

Supplement: Supplementary file 3 [file Image2.TIF]

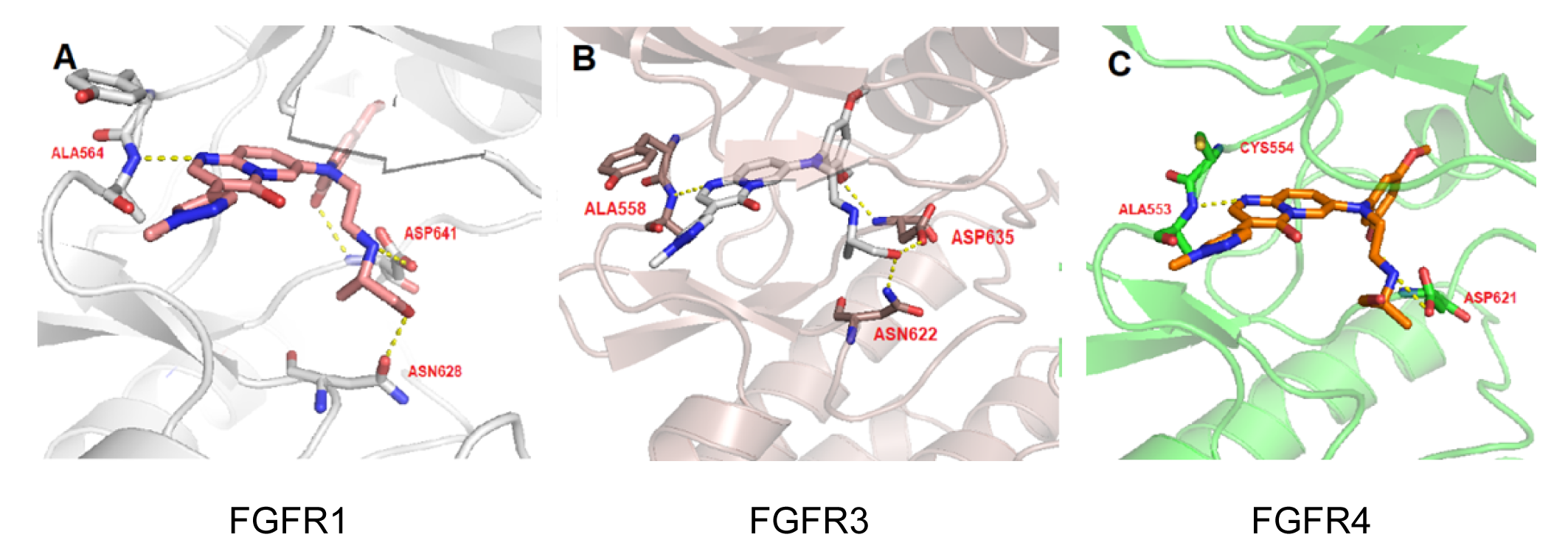

Supplement: Supplementary file 4 [file Image1.TIF]
